# Constitutive proteins of lumpy skin disease virion assessed by next-generation proteomics

**DOI:** 10.1128/jvi.00723-23

**Published:** 2023-09-22

**Authors:** Léo Schlosser-Perrin, Philippe Holzmuller, Bernard Fernandez, Guylaine Miotello, Noureddine Dahmani, Aymeric Neyret, Stéphane Bertagnoli, Jean Armengaud, Philippe Caufour

**Affiliations:** 1 UMR ASTRE, CIRAD, INRAE, University of Montpellier (I-MUSE), Montpellier, France; 2 Département Médicaments et Technologies pour la Santé, Université Paris Saclay, CEA, INRAE, Bagnols-sur-Cèze, France; 3 CEMIPAI, University of Montpellier, UAR3725 CNRS, Montpellier, France; 4 IHAP, Université de Toulouse, INRAE, ENVT, Toulouse, France; Northwestern University Feinberg School of Medicine, Chicago, Illinois, USA

**Keywords:** lumpy skin disease, mature virion, viral particle proteome, viral proteins, Capripoxvirus

## Abstract

Lumpy skin disease (LSD) virus, a ruminant poxvirus of the *Capripoxvirus* genus, is the etiologic agent of an economically important cattle disease categorized as a notifiable disease by the World Organisation for Animal Health. The large and complex enveloped LSD virus (LSDV) particle encloses a double-stranded linear DNA genome of 151 kbp, comprising 156 predicted ORFs, together with a variety of proteins that are not yet identified. In parallel with a recent widespread expansion of LSD, many LSDV whole genomes have been sequenced, but knowledge on protein composition of the LSDV particle remains missing. In this study, LSD mature virions (MV) from strain KSGP-0240 were purified through a multistep ultracentrifugation process. The protein composition of LSD virions was then analyzed using label-free shotgun proteomics, based upon nano-liquid chromatography (LC) and tandem mass spectrometry. This procedure resulted in the identification of a total of 111 LSDV proteins. Considering that this first MV proteome extended beyond packaged proteins into the field of contaminants, an analytical methodology was developed and made it possible to select 66 viral proteins as candidates for packaging into MV. These viral proteins were analyzed comparatively with proteins previously demonstrated to be constitutive of the vaccinia virus MV particle. Offering for the first time a comprehensive proteomic analysis of an LSDV strain, this study contributes to our understanding of the structural features of infectious LSDV MV particles and paves the way for further systematic proteomic characterization of other LSDV strains.

**IMPORTANCE:**

*Lumpy skin disease virus* (LSDV) is the causative agent of an economically important cattle disease which is notifiable to the World Organisation for Animal Health. Over the past decades, the disease has spread at an alarming rate throughout the African continent, the Middle East, Eastern Europe, the Russian Federation, and many Asian countries. While multiple LDSV whole genomes have made further genetic comparative analyses possible, knowledge on the protein composition of the LSDV particle remains lacking. This study provides for the first time a comprehensive proteomic analysis of an infectious LSDV particle, prompting new efforts toward further proteomic LSDV strain characterization. Furthermore, this first incursion within the capripoxvirus proteome represents one of very few proteomic studies beyond the sole *Orthopoxvirus* genus, for which most of the proteomics studies have been performed. Providing new information about other chordopoxviruses may contribute to shedding new light on protein composition within the *Poxviridae* family.

## INTRODUCTION

The *Poxviridae* (POXV) is a family of large, complex, enveloped virions enclosing single linear double-stranded DNA genome (128–450 kbp) (1, 2) that replicates entirely in the cytoplasm of a wide range of vertebrate or invertebrate cells (2). The viral particles enclose proteins with a variety of functions including structural proteins and enzymes involved in the early steps of virus infection and DNA repair (2). The viral infection cycle produces sequentially multiple forms of infectious viral particles, all of which share the same mature virus (MV) at their center (3, 4). Poxviral morphogenesis has been extensively characterized for the laboratory prototype virus used for the study of poxvirus, the vaccinia virus (VACV) (5).

Vaccinia (VAC) MV virions, the first infectious form produced during the infection course, remain mostly within the cell until lysis. As the most basic and the most abundant infectious form, VAC MV virions have been used for the vast majority of experimental studies. After its assembly inside cytoplasm, a portion of VAC MV is wrapped with two additional membrane layers of Golgi membrane to form intracellular enveloped virion or wrapped virion (WV). VAC WV is then transported to the cell plasma membrane where it loses one membrane during virion egress to become a cell-associated extracellular enveloped virion (CEV). The VAC CEV may remain associated to the cell surface or can be released into the medium to become an extracellular enveloped virion (EEV). The major infectious forms of VACV, MV, and extracellular virions (EVs), namely CEV and EEV, display different biological and immunological properties (5) associated to their different roles in VACV pathogenesis. MV is robust and known to resist environmental and physical changes, whereas EVs are very fragile and the integrity of their outer membranes can be altered during purification procedures (6 – 8). EVs are thought to be involved in cell-to-cell spread within an organism, while MVs are considered to mediate long-range dissemination and transmission between hosts in the environment (2, 5).

POXV is divided into the subfamilies *Chordopoxvirinae* and *Entomopoxvirinae* based on vertebrate and arthropod host range (2). The subfamily *Chordopoxvirinae* consists of 18 genera, among which the *Capripoxvirus* (CaPV) genus comprises three members: *sheeppox virus* (SPPV), *goatpox virus* (GTPV), and *lumpy skin disease virus* (LSDV) (2). LSDV is responsible for an economically important disease in cattle and Asian water buffalos (*Bubalus bubalis*), which is notifiable to the World Organisation for Animal Health (WOAH). Over the past decades, this disease has spread around the world at an alarming rate throughout most of the African continent, the Middle East, the Balkan region, the Caucasus, Kazakhstan, parts of the Russian Federation, and recently affecting many Asian countries (9 – 20). The different measures to control and eradicate LSD include the early detection of outbreaks, feasible stamping out policy, quarantine, trade and movement restrictions, and vector control as well as vaccination relying mostly on homologous attenuated LSDV vaccine strains (21).

With the recent expansion of LSD, multiple sequences of whole genomes have been made available from different affected countries, including virulent field strains and attenuated vaccine strains. Despite a very high level of sequence identity at the genomic level [at least 98% (22 – 25)], some strains may exhibit distinct *in vitro* and *in vivo* biological patterns. Considering the information gaps existing between strains, an important characterization effort must be made, especially in terms of molecular description such as protein composition and interactions.

Among the viral strains whose genome was recently fully sequenced, the attenuated vaccine KSGP-0240 strain draws specific attention (26, 27). Longtime considered as an SPPV strain, this strain proved to be actually an LSDV strain (26 – 29). Displaying only a two-nucleotide difference with the NI-2490 Neethling field strain (27), this vaccine strain is phylogenetically grouped within virulent field strains (30). Its parental wild-type strain was isolated in the field from a sheep during a sheep and goat pox outbreak in a mixed flock (31). Such an isolation of an LSDV strain from naturally infected sheep in the field is, to our knowledge, a very unique situation deserving the greatest attention. The parental wild-type isolate was passed only a limited number of times to obtain the KSGP-0240 vaccine strain (32). Finally, the poor biological characterization of this vaccine strain is illustrated through the limited number of well-designed studies addressing properly the *in vivo* characterization of the KSGP-0240 strain (33 – 38).

Owing to these very unique traits, we decided to start our characterization of LSDV proteome with the KSGP-0240 strain. Viral stocks were produced on Madin-Darby bovine kidney (MDBK) cells and purified following a multistep purification procedure, including, in particular, different continuous gradients (either tartrate or sucrose). To obtain a comprehensive list of viral and host proteins comprising each LSD MV virion, the purified viral fractions were analyzed through a label-free shotgun approach, based on nano-liquid chromatography of the peptides and their analysis with high-resolution tandem mass spectrometry (MS). In this experiment, a total of 111 viral proteins and 1,473 host proteins were identified. To discriminate the specifically packaged proteins from the contaminants, an analytical strategy was developed, taking advantage of two density gradient media (tartrate versus sucrose) used through the purification workflow. Applying this analytical methodology on the whole set of identified proteins, we finally proposed 66 viral proteins as constitutive of the MV virion of the KSGP-0240 strain. In addition, of the 1,473 host proteins, 65 proteins were identified as potential candidates for packaging. The selected viral proteins were grouped within certain functional categories (e.g., cell attachment/entry, viral transcription, structural proteins, and genome integrity) and analyzed comparatively with proteins previously identified as selectively packaged in the VACV particle (39, 40). Altogether, our results offer for the first time a comprehensive proteomic analysis of an LSD strain, paving the way for further systematic proteomic characterization of other LSDV strains.

## MATERIALS AND METHODS

### Virus and cell lines

The LSD virus strain KSGP-0240 was obtained from commercial producer JOVAC (Jordan Bio Industries Center, Amman, Jordan) and replicated in MDBK cells (NBL1, ATCC CCL22, Manassas, United States). MDBK cell lines were grown in Dulbecco’s Modified Eagle Medium (Eurobio Scientific, Les Ulis, France) and supplemented with Eagle’s non-essential amino acid (Eurobio Scientific, Les Ulis, France), L-glutamine (Eurobio Scientific, Les Ulis, France), sodium pyruvate (Eurobio Scientific, Les Ulis, France), and 5% fetal bovine serum (Dutscher, Bernolsheim, France).

### Virus production and purification

To prepare the viral production batches, the KSGP-0240 strain was passed three times in MDBK cells after resuspension of the freeze-dried lyophilizate (Jordan Bio Industries Center, Amman, Jordan). Near confluent monolayers of MDBK cells were infected with LSDV at a multiplicity of infection (MOI) of 0.2. After 48 hours, the infected cells can be processed as described below when they have reached around 60% confluence, and a cytopathic effect (CPE) is evenly distributed around the monolayer. The viruses were then purified as described in previous studies, with some modifications of the original protocol (40 – 44). In brief, cells were harvested when the cytopathic effects were moderate (around 40% of the monolayer is lysed). The supernatant was discarded. The cell monolayer was frozen and thawed three consecutive times and then cellular debris pelleted at 400 g for 15 minutes. The supernatant was thereafter centrifuged at 80,000 g for 1 hour at 4°C (Beckman Optima L70, SW28). The medium was discarded and the virus pellet resuspended in PBS. The viral suspension was then sonicated (Bioblock scientific ultrasonic processor, 20 W, 40 seconds), incubated successively with DNase for 15 minutes, then with trypsin for 15 minutes, and finally sonicated again (Bioblock scientific ultrasonic processor, 20 W, 40 seconds). The viral suspension was subsequently layered on top of a double sucrose cushion of 36%–72% and centrifuged at 100,000 g for 1 hour 15 minutes (Beckman Optima L70, SW41). The visible band of virion at the interface of the sucrose cushion at 72% and 36% was collected through pinholes. Finally, the supernatant was pelleted at 150,000 g for 1 hour (Beckman Optima L70, SW41), resuspended with Tris-EDTA solution, pH 7 (Tris-HCL 50 mM, EDTA 1 mM), and stored overnight at 4°C. The collected virus was then split into two halves. The first half was placed on a continuous sucrose gradient, ranging from 60% to 40% and centrifuged at 58,000 g for 75 minutes (Beckman, Optima L70, SW41). The other half was placed on a continuous tartrate gradient, ranging from 41% to 8% and centrifuged at 150,000 g for 1 hour 30 minutes (Beckman, Optima L70, SW41). In the sucrose gradient, the viruses usually formed two diffuse bands about two-thirds of the way down the tube; on occasions, another band was obtained one-third of the way down the tube. All bands were collected through pinholes, using a sterile syringe, pooled and centrifuged at 150,000 g for 1 hour (Beckman, Optima L70, SW41) before resuspension in 2-D differential in-gel electrophoresis(DIGE) solution (7 M urea, 2 M thiourea, 4% CHAPS, 30 mM Tris Hcl, 0.5% Triton, and complete protease inhibitor cocktail) (45) for final storage. In the tartrate continuous gradient, the virus formed a single band which had to be collected through pinholes using a sterile syringe. This band was centrifuged at 150,000 g for 1 hour (Beckman, Optima L70, SW41) before resuspension in DIGE solution (45) for final storage.

### Protein quantification with micro-Bradford

Pierce 660-nm Protein Assay Reagent (ThermoFisher Scientific, Invitrogen, Waltham, MA, USA) was used for measuring protein concentrations, according to the supplier’s specifications for microplate procedure.

### Viral DNA isolation, PCR and sequencing

DNA was extracted and purified, either from infected MDBK cells or from water-dissolved lyophylizate of the KSGP-0240 vaccine vial, following the manufacturer’s instructions (Quick-DNA/RNA-Viral Kit, Zymo research Corp., Tustin, USA). In order to characterize the presence of capripoxviruses in the extracted DNA, DNA-purified samples were first analyzed using gel-based PCR methods described previously (46, 47). Further characterization was then undertaken targeting specifically LSDV genes of interest through PCR: LSDV 011 (28), LSDV 049 (forward primer: ACCTCAATCAAAGGAACTATGGCA, reverse primer: CCTTTTCTTTGTTCCCGCATAGA), and LSDV 134 (forward primer: TCGTCTGATAGCGGCATTGT, reverse primer: TTGGTGATTAGCCTGTGCCA). Thereafter, the corresponding PCR products were Sanger sequenced (Genewiz-Azenta, Leipzig, Germany), and sequence analyses were performed using Geneious software, version 10.2.6 (Biomatters, Newton, New Zealand).

### Semi-quantitative determination of viral DNA using real-time PCR

After purification of virion and lysis in the manufacturer’s lysis solution, samples were processed according to the manufacturer’s instructions (ThermoFisher Scientific, Invitrogen, Waltham, MA USA), and semi-quantitative PCR was performed as described in the application note (“Real-Time PCR Using Platinum Direct PCR Universal Master Mix”) supplied with the kit’s manual referred to above. The H3L primers (H3L forward: AAAACGGTATATGGAATAGAGTTGGAA, H3L reverse: AAATGAAACCAATGGATGGGATA) used were developed previously (48).

### SDS-PAGE

Viral purified extracts, resuspended and stored in DIGE solution, were applied to a 4%–12% Bis-Tris gel (Invitrogen, Carlsbad, USA), and a constant voltage of 70 V was applied for stacking and then a constant voltage of 200 V for migration in NuPage MES-SDS running buffer (Invitrogen, Carlsbad, USA). Then the gel was stained with SimplyBlue SafeStain (Life technologies, Carlsbad, USA) and washed three times with Milli-Q water.

### Virus titration

Viral stocks of KSGP-0240 were titrated as previously described, following WOAH recommendations (49). MDBK cells were used for the test and cultured in 96-well flat-bottomed tissue-culture grade microtiter plates. The plates were incubated at 37°C, 5% carbon dioxide (CO_2_) for 9 days. The plates were examined under an inverted microscope for the presence of a CPE starting from day 4. The final reading, taken on day 9, is used to determine the titer, which is calculated using the Kärber method (49).

### Electron microscopy

Samples (viral particles purified from sucrose gradient or from tartrate gradients) were fixed with 2.5% (vol/vol) glutaraldehyde in PHEM (PIPES, HEPES, EGTA and MgCl2) buffer and post-fixed in osmium tetroxide 1%/K_4_Fe (CN)_6_ 0.8%, at room temperature for 1 hour. The samples were then dehydrated in successive ethanol baths (50/70/90/100%) and infiltrated with propylene oxide/EMbed812 mixes before embedding. Seventy nanometer ultrathin cuts were made on a PTXL ultramicrotome (RMC, France), stained with uranyl acetate/lead citrate, and observed on a Tecnai G2 F20 (200 kV, FEG) TEM at the Electron Microscopy Facility COMET, INM, Montpellier.

### Proteomics

Extracted proteins denatured in the lysis buffer were digested in gel with Trypsin Gold (V5280, Promega, Madison, USA) using 0.011% ProteaseMAX surfactant (V2071, Promega, Madison, USA) (50, 51). The resulting peptides were quantified using the Pierce Quantitative Fluorometric Peptide Assay and then a quantity of 220 ng was resolved on an UltiMate 3000 NanoLC chromatography system coupled to a Q-Exactive HF mass spectrometer (ThermoFisher Scientific, llkirch-Graffenstaden Les Ulys, France) operated as previously described (52) for analysis. The peptides were desalted on an Acclaim PepMap100 C18 precolumn (5 µm particle size, 100 Å pore size, 300 µm id × 5 mm) and then resolved according to their hydrophobicity on a nanoscale Acclaim PepMap 100 C18 column (3 µm particle size, 100 Å pore size, 75 µm id × 50 cm) at a flow rate of 200 nL/minute. The gradient was developed from 4% to 22% of CH3CN supplemented with 0.1% formic acid over 50 minutes and then from 10% to 32% over 20 minutes. Mass spectrometry was performed in a data-dependent acquisition mode following a Top20 strategy with full MS scans acquired from 350 to 1,500 m/z at a 60,000 resolution. After each scan of precursors, the 20 most abundant ions were sequentially selected for fragmentation and MS/MS acquisition at a 15,000 resolution. An intensity threshold of 8.3 × 10^4^ was applied. A 10-second dynamic exclusion was used to increase the detection of low-abundance peptides. Only double- and triple-charged ions were selected for MS/MS analysis.

The MS/MS data were searched against LSDV and *Bos taurus* protein sequences (127,411 sequences) using Mascot software, version 2.6.1 (Matrix Science, Boston, USA). The search parameters included only 2+ and 3+ peptide charges, 5 ppm mass tolerance for the parent ion, 0.02 Da mass tolerance for MS/MS ions, and a maximum of two missed cleavage sites for trypsin. For the database search, the carbamidomethyl (C) modification was considered as a fixed modification, and the following variable modifications were considered: oxidation (M) and deamidation (NQ). All peptide matches with a peptide score associated with a Mascot *P*-value of less than 0.05 were retained. Proteins were considered valid when at least two distinct peptides were detected in the same sample. The MS proteomics data have been deposited to the ProteomeXchange Consortium via the PRIDE partner repository with the data set identifier PXD037293 and 10.6019/PXD037293.

Protein abundance was estimated using the normalized spectral abundance factor (NSAF) as previously described (53). The NSAF for each protein was calculated by dividing their spectral counts (SpC) by their molecular mass expressed in kilodalton. The percentage of NSAF provides a measure of relative abundance, making it possible to compare the abundance between different proteins within the same sample.

## RESULTS

### Purification and quality control of LSDV particles

**Fig 1:**
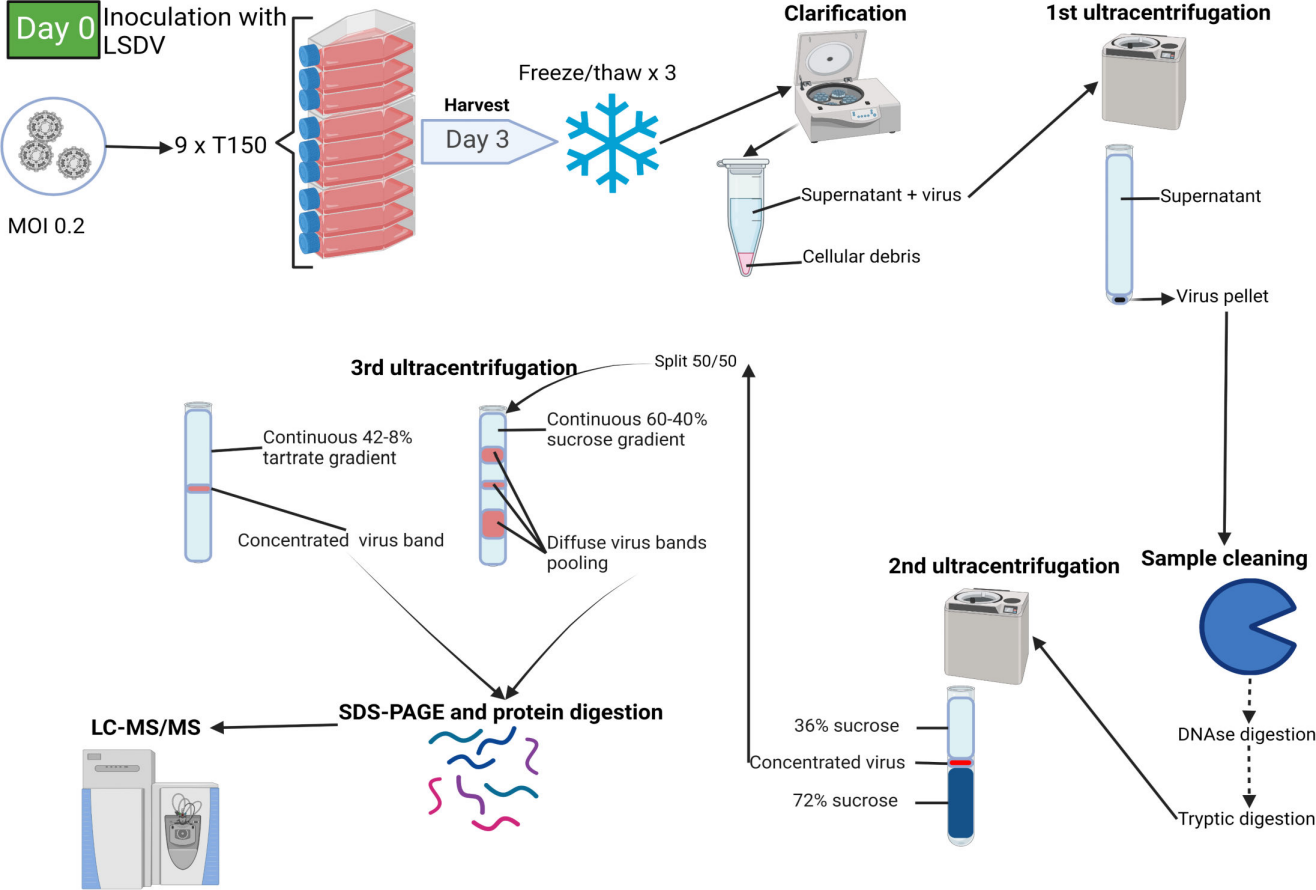
Schematic representation of the experimental design. Schematic representation of viral production and purification workflow from cell culture to mass spectrometry. MDBK cells were infected with the LSDV 0240 strain (MOI: 0.2). LSDV virion is thereafter purified through serial centrifugation. Purified viral extracts resuspended and lysed in DIGE buffer are finally analyzed using nano-LC and tandem MS. The main experimental steps and the output of the analysis are highlighted.

Figure 1 shows the experimental workflow adopted to decipher the proteome of LSDV particles. The viral particles were obtained from infected MDBK cells and purified either on sucrose or tartrate continuous gradients. Continuous sucrose gradients allow nonequilibrium rate zonal velocity sedimentation to be used, while continuous tartrate gradients allow for equilibrium buoyant density isopycnic banding.

First, the identity of the virus strain provided by the manufacturer, namely LSDV KSGP-0240, was confirmed using PCR and sequencing (data not shown).

**Fig 2:**
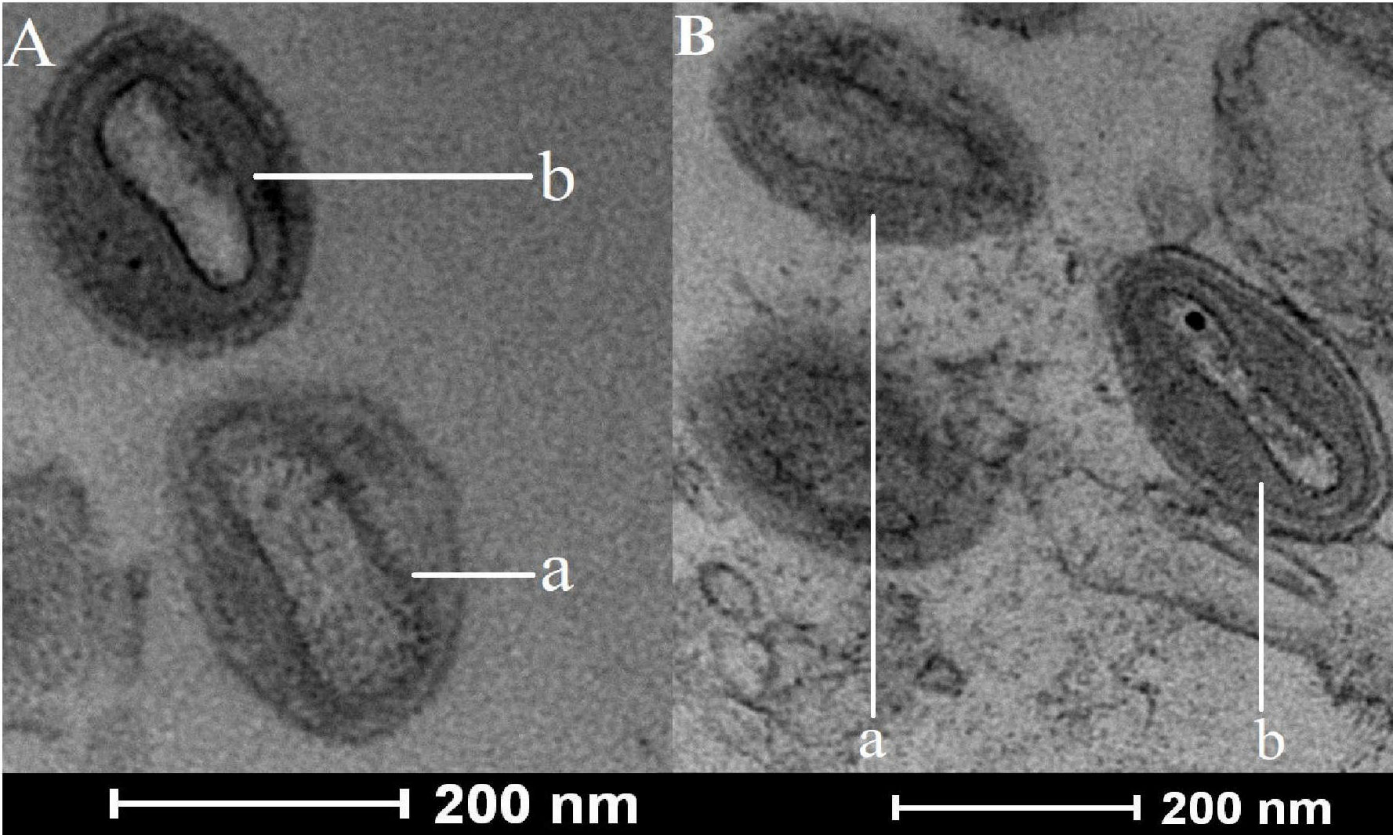
LSDV particles observed in purified virus preparations. (**A**) Sucrose-purified LSDV particles were analyzed using transmission electron microscopy. Both particle types are present: (**a**) mature virus particles and (**b**) enveloped virus particles. (**B**) Tartrate-purified LSDV particles under transmission electron microscopy with (**a**) mature virus particles and (**b**) enveloped virus particles.

At the development stage of both methods, virions collected from the sucrose or tartrate bands were tested for infectiousness (titration) and morphology (electron microscopy). Titration of these viral suspensions demonstrated that purified LSDV particles were still infectious (data not shown). As indicated in EM micrographs (Fig. 2), well-organized viral particles were visible in both conditions (tartrate and sucrose). Elongated brick-shaped viral particles were observed, and, in some fields, even lateral bodies were visible. The two highly organized brick-shaped viral forms were observed with two (EV) or one membrane (MV) layers. Contaminant membranes, cellular organelles, and debris were also identified.

At the production stage of replicates for MS analyses, in order to assess the variability between replicates, samples of five replicates were taken at two steps of the LSDV purification procedure and then processed for quality control by real-time PCR and viral titration. The first sampling was performed at the start of the workflow, immediately after cell culture lysis and discarding of the supernatant. The second sampling in the workflow was performed at the end of the procedure, immediately after viral resuspension with DIGE buffer solution. No significant difference in genome copy number or in viral titer was demonstrated between the five replicates of each of the two virion purification conditions (Table S1).

Finally, by considering NSAF as an approximate surrogate of protein abundance, it is possible to compare the protein quantities between replicates. For each replicate, the NSAF score is used as a relative quantification method, representing a more refined version compared to simple spectral counting. Comparison of protein quantities between replicates for each of the two virion purification conditions (tartrate or sucrose) confirmed the limited variability highlighted by the above parameters (viral titer, genome copy number) (Table S1).

### Proteome of the lumpy skin disease virion preparation

The proteins of the purified viral extracts (five replicates for each of the two virion purification conditions) were digested with trypsin, and the resulting peptides were identified independently using high-resolution tandem MS.

A total of 15,974 specific MS/MS spectra made it possible to identify 111 LSDV proteins, certified with high confidence. Out of 156 *in silico* predicted coding sequences annotated on the LSDV genome (26, 27), 111 LSDV proteins were identified at least once across all replicates. We noted an average of 14 specific peptides for each protein with a range of 2 to 91 specific peptides. The peptide coverage varies from 92% to 4% with the best covered proteins being LSDV 28, 31, 34, 53, 63, 95, and 115 (>80% coverage).

In addition, NSAF data regarding the five replicates of the two virion purification conditions show that 25% (mean of total NASF for viral protein in sucrose) and 30% (mean of total NASF for viral protein in tartrate) of the signal were assigned to viral proteins in the corresponding viral preparations (Table S1). However, it should be noted that the dynamic exclusion threshold used for selecting peptides for fragmentation actually decreases the count of the most abundant proteins, therefore reducing the viral protein ratio by a significant factor (54, 55).

A thorough description of these proteins is provided in Table 1. In order to assign functions to proteins identified through our study, we relied on the genomic annotation work performed previously (26, 27), taking advantage of comparison-based prediction with viruses of the same POXV family such as VACV and myxoma virus (MYXV). Finally, Table 1 proposes the corresponding LSDV gene number and, when identified in previous studies (26, 27), VACV and MYXV orthologs.

**TABLE 1:** LSDV detected and “packaged” proteins by LC-MS/MS^*d*^

LSDV gene	ORF-VACV^*a*^	ORF-MYXV^*b*^	Functional description	Mass	Specific peptides	Total peptides	Numbers of hits across replicates	Rank (relative abundance)	Sucrose (NSAF)	Tartrate (NSAF)	Sum of NSAF	Protein accession no.^*c*^	Packaged protein
LSDV001	B15R	M003.1	Hypothetical protein	17,846	4	6	4	82	0.5043	1.0086	1.5129	AOE47577.1	YES
LSDV003	B9R	M004	Putative ER-localized apoptosis regulator	27,443	9	12	3	53	1.6033	2.1499	3.7532	AOE47579.1	YES
LSDV004		M004.1	Hypothetical protein	6,735	8	8	3	14	6.8300	8.3148	15.1448	AOE47580.1	YES
LSDV007	C10L		Hypothetical protein	41,881	9	12	3	89	0.3582	0.6208	0.9790	AOE47583.1	YES
LSDV012	B4R	M149R	Ankyrin repeat protein	24,876	4	5	3	87	0.2814	0.7638	1.0452	AOE47588.1	YES
LSDV013	B16R		Interleukin-1 receptor-like protein S78201	40,106	5	8	3	104	0.2992	0.2743	0.5735	AOE47589.1	YES
LSDV017		M011L	Anti-apoptotic membrane protein	20,685	5	6	4	73	0.7735	1.5954	2.3689	AOE47593.1	YES
LSDV018	F2L	M012L	DUTPase	16,175	7	7	5	26	2.4730	4.0804	6.5533	AOE47594.1	YES
LSDV028	F13L	M022L	Palmytilated EEV membrane glycoprotein	41,208	31	35	5	9	10.7018	20.3844	31.0862	AOE47604.1	YES
LSDV031	F17L	M026L	DNA-binding virion core protein	11,625	12	12	3	1	24.2581	54.2796	78.5376	AOE47607.1	YES
LSDV037	E6R	M032R	Hypothetical protein	67,160	23	27	4	52	1.5932	2.3079	3.9011	AOE47613.1	YES
LSDV038	E8R	M033R	Putative membrane protein	31,363	11	11	5	43	1.7218	2.8696	4.5914	AOE47614.1	YES
LSDV040	E10R	M035R	Sulfhydryl oxidase	11,278	3	3	3	72	0.7980	1.5960	2.3940	AOE47616.1	YES
LSDV041	E11L		Putative virion core protein	15,087	7	9	4	38	1.7233	3.7118	5.4351	AOE47617.1	YES
LSDV042	O1L	M036L	Hypothetical protein	79,073	24	32	4	67	1.1761	1.6820	2.8581	AOE47618.1	YES
LSDV043	I1L	M038L	Putative DNA-binding virion core protein	36,509	21	24	5	33	2.2186	3.6155	5.8342	AOE47619.1	YES
LSDV044	12L	M039L	Hypothetical protein	8,468	3	3	3	48	1.4171	2.7161	4.1332	AOE47620.1	YES
LSDV045	I3L	M040L	DNA-binding phosphoprotein	31,061	16	18	5	30	2.4146	3.5736	5.9882	AOE47621.1	YES
LSDV050	G1L	M045L	Putative metalloprotease	69,181	14	22	4	62	0.8673	2.2405	3.1078	AOE47626.1	YES
LSDV052	G3L	M046L	Hypothetical protein	12,955	7	7	3	58	0.8491	2.4701	3.3192	AOE47627.1	YES
LSDV053	G4L	M048L	Glutaredoxin-like protein	14,395	10	11	4	16	3.7513	7.6415	11.3928	AOE47629.1	YES
LSDV057	G7L	M052L	Putative virion core protein	42,031	20	26	3	21	2.3078	4.5918	6.8997	AOE47633.1	YES
LSDV058	G8R	M053R	Late transcription factor VLTF-1	30,026	11	14	4	50	1.6319	2.4312	4.0631	AOE47634.1	YES
LSDV059	G9R	M054R	Poxvirus myristoylprotein	38,917	10	12	5	77	0.6938	1.2334	1.9272	AOE47635.1	YES
LSDV060	L1R	M055R	Putative myristylated IMV envelope protein	26,830	10	14	3	28	2.0127	4.3235	6.3362	AOE47636.1	YES
LSDV063	L4R	M058R	DNA-binding virion core protein	29,017	32	35	4	2	19.6437	41.7686	61.4123	AOE47639.1	YES
LSDV064	L5R	M059R	Putative membrane protein	15,475	9	10	3	25	2.3263	4.2649	6.5913	AOE47640.1	YES
LSDV065	J1R	M060R	Hypothetical protein	17,370	7	7	3	69	0.7484	1.8423	2.5907	AOE47641.1	YES
LSDV069	J4R	M066R	RNA polymerase subunit	21,198	8	9	3	49	1.1794	2.9248	4.1042	AOE47645.1	YES
LSDV070	J5L	M067L	Hypothetical protein	15,421	3	6	4	75	0.6485	1.4266	2.0751	AOE47646.1	YES
LSDV071	J6R	M068R	RNA polymerase subunit	147,339	71	92	3	20	3.0406	4.1808	7.2214	AOE47647.1	YES
LSDV072	H1L	M069L	Putative protein-tyrosine phosphatase	19,938	16	17	5	13	7.0719	14.3445	21.4164	AOE47648.1	YES
LSDV073	H2R	M070R	Putative viral membrane protein	22,096	8	9	4	66	0.9051	1.9913	2.8965	AOE47649.1	YES
LSDV074	H3L	M071L	Putative IMV envelope protein	37,528	35	40	3	7	17.7734	23.5291	41.3025	AOE47650.1	YES
LSDV079	D1R	M076R	MRNA capping enzyme small subunit	98,889	41	59	3	31	2.4674	3.4483	5.9157	AOE47655.1	YES
LSDV080	D2L	M077L	Hypothetical protein	18,125	9	11	3	45	1.2138	3.2000	4.4138	AOE47656.1	YES
LSDV081	D3R	M078R	Putative virion protein	29,578	15	18	3	39	1.9609	3.3809	5.3418	AOE47657.1	YES
LSDV083	D5R	M080R	Putative NTPase	90,813	16	20	4	83	0.4184	0.7488	1.1672	AOE47659.1	YES
LSDV084	D6R	M081R	Putative early transcription factor small subunit	73,386	21	27	3	63	1.2945	1.8123	3.1069	AOE47660.1	YES
LSDV089	D12L	M087L	MRNA capping enzyme small subunit	33,515	16	18	3	32	2.0886	3.8192	5.9078	AOE47665.1	YES
LSDV090	D13L	M088L	Putative rifampicin resistance protein	62,156	17	20	3	74	1.0618	1.2710	2.3328	AOE47666.1	YES
LSDV091	A1L	M089L	Late transcription factor VLTF-2	16,821	7	8	4	60	0.9512	2.3185	3.2697	AOE47667.1	YES
LSDV093	8.9kd	M091L	Hypothetical protein	9,017	4	4	3	80	0.3327	1.2199	1.5526	AOE47669.1	YES
LSDV094	A3L	M092L	Putative virion core protein	75,544	49	53	5	4	21.0076	35.2907	56.2983	AOE47670.1	YES
LSDV095	A4L	M093L	Virion core protein	18,666	23	32	3	10	10.4468	18.0006	28.4474	AOE47671.1	YES
LSDV097	A6L	M095L	Hypothetical protein	43,650	18	24	5	44	1.5578	2.8866	4.4444	AOE47673.1	YES
LSDV098	A7L	M096L	Putative early transcription factor large subunit	82,825	40	48	3	24	2.7166	3.8998	6.6164	AOE47674.1	YES
LSDV101	A10L	M099L	Putative virion core protein	104,152	91	96	3	3	23.3505	36.5908	59.9412	AOE47677.1	YES
LSDV102	A11R	M100R	Hypothetical protein	36,016	7	8	3	84	0.3054	0.8052	1.1106	AOE47678.1	YES
LSDV104	A13L	M102L	IMV membrane protein	7,743	4	4	4	15	4.7785	8.9113	13.6898	AOE47680.1	YES
LSDV105	A14L	M103L	IMV membrane protein	10,250	6	6	3	6	19.1220	25.8537	44.9756	AOE47681.1	YES
LSDV107	A15L	M105L	Hypothetical protein	11,194	4	5	3	41	1.3400	3.3947	4.7347	AOE47683.1	YES
LSDV108	A16L	M106L	Putative myristylated membrane protein	43,909	21	27	3	35	2.3458	3.2340	5.5797	AOE47684.1	YES
LSDV111	A19L	M109L	Hypothetical protein	8,162	3	3	3	76	0.9802	0.9802	1.9603	AOE47687.1	YES
LSDV113	A21L	M110L	IMV membrane protein	13,550	7	7	4	29	2.1402	3.9852	6.1255	AOE47688.1	YES
LSDV117	A27L	M115L	Hypothetical protein	17,389	22	27	3	11	8.4536	15.5846	24.0382	AOE47693.1	YES
LSDV118	A28L	M116L	Hypothetical protein	16,192	4	5	3	65	0.9881	2.0380	3.0262	AOE47694.1	YES
LSDV119	A29L	M117L	RNA polymerase subunit	35,214	11	11	3	64	1.2779	1.7891	3.0670	AOE47695.1	YES
LSDV121	A32L	M120L	Putative DNA packaging protein	29,081	9	11	3	54	1.1004	2.6478	3.7482	AOE47697.1	YES
LSDV131	A45R	M131R	Superoxide dismutase-like protein	17,677	8	9	3	42	1.2446	3.4508	4.6954	AOE47707.1	YES
LSDV132			Hypothetical protein	20,399	2	3	4	106	0.2451	0.2451	0.4902	AOE47708.1	YES
LSDV133	A50R	M133R	DNA ligase-like protein	64,562	7	9	3	101	0.1549	0.4492	0.6041	AOE47709.1	YES
LSDV137	A51R	M137R	Hypothetical protein	38,374	14	17	4	61	1.3811	1.8763	3.2574	AOE47712.1	YES
LSDV139	B1R	M142R	Putative Ser/Thr protein kinase	35,612	5	9	4	96	0.2246	0.5335	0.7582	AOE47714.1	YES
LSDV144	A55R	M140R	Kelch-like protein	64,588	2	3	3	110	0.0929	0.0929	0.1858	AOE47719.1	YES
LSDV148	B4R	M148R	Ankyrin-like protein	52,230	8	11	3	88	0.3255	0.7084	1.0339	AOE47723.1	YES
LSDV002		M003.2	Hypothetical protein	15,477	15	15	2	8	17.6391	21.9035	39.5425	AOE47578.1	NO
LSDV006	B16R		IL-1 receptor-like protein	27,351	14	17	2	47	2.0109	2.3400	4.3508	AOE47582.1	NO
LSDV008	B8R	M007	Putative soluble interferon gamma receptor	31,845	8	10	1	103	0.2826	0.3140	0.5966	AOE47584.1	NO
LSDV011			G protein-coupled chemokine receptor-like protein	43,378	3	3	0	109	0.2997	0.1153	0.4150	AOE47587.1	NO
LSDV015			IL-18 binding protein	18,839	5	6	2	86	0.7962	0.2654	1.0616	AOE47591.1	NO
LSDV024	F9L	M019L	S-S bond formation pathway protein	24,865	4	5	1	102	0.1207	0.4826	0.6033	AOE47600.1	NO
LSDV025	F10L	M020L	Ser/Thr kinase	53,290	18	19	1	57	1.5763	1.8390	3.4153	AOE47601.1	NO
LSDV027	F12L	M021L	EEV maturation protein	74,818	12	16	2	108	0.2272	0.2272	0.4544	AOE47603.1	NO
LSDV032	E1L	M027L	Poly(A) polymerase large subunit	54,531	28	30	2	22	2.5857	4.2178	6.8035	AOE47608.1	NO
LSDV033	E2L	M028L	Hypothetical protein	86,539	12	15	1	111	0.0462	0.0231	0.0693	AOE47609.1	NO
LSDV034	E3L	M029L	Double-strand RNA-binding protein	20,656	18	21	2	12	9.9729	14.0395	24.0124	AOE47610.1	NO
LSDV039	E9L	M034L	DNA polymerase	117,411	55	65	0	55	2.1293	1.5842	3.7135	AOE47615.1	NO
LSDV047	I6L	M042L	Putative DNA-binding protein	46,122	8	10	1	85	0.6504	0.4553	1.1058	AOE47623.1	NO
LSDV048	I7L	M043L	Putative virion core protein	50,753	22	26	2	27	2.8570	3.5860	6.4430	AOE47624.1	NO
LSDV049	I8R	M044R	RNA helicase NPH-II	78,231	22	25	2	68	1.2399	1.3933	2.6332	AOE47625.1	NO
LSDV055	G5.5R	M050R	RNA polymerase	7,245	4	4	2	56	0.9662	2.4845	3.4507	AOE47631.1	NO
LSDV056	G6R	M051R	Hypothetical protein	19,550	4	4	0	100	0.2046	0.4092	0.6138	AOE47632.1	NO
LSDV062	L3L	M057L	Hypothetical protein	37,386	16	19	2	18	4.0657	5.4566	9.5223	AOE47638.1	NO
LSDV066	J2R	M061R	Thymidine kinase	20,434	2	3	2	107	0.0979	0.3915	0.4894	AOE47642.1	NO
LSDV068	J3R	M065R	Poly(A) polymerase small subunit	39,415	21	23	2	19	2.9684	5.0488	8.0173	AOE47644.1	NO
LSDV075	H4L	M072L	RNA polymerase-associated protein	94,726	41	48	2	37	2.3542	3.1354	5.4895	AOE47651.1	NO
LSDV076	H5R	M073R	Late transcription factor VLTF-4	25,092	5	8	0	81	0.9166	0.5978	1.5144	AOE47652.1	NO
LSDV077	H6R	M074R	DNA topoisomerase type I	37,148	17	21	2	51	1.7767	2.2074	3.9841	AOE47653.1	NO
LSDV078	H7R	M075R	Hypothetical protein	17,259	4	4	2	79	0.5215	1.1009	1.6223	AOE47654.1	NO
LSDV085	D7R	M082R	RNA polymerase subunit	18,371	3	3	0	93	0.0544	0.8165	0.8709	AOE47661.1	NO
LSDV088	D11L	M086L	Putative transcription termination factor	73,615	32	42	1	40	2.4316	2.8798	5.3114	AOE47664.1	NO
LSDV096	A5R	M094R	RNA polymerase subunit	19,499	7	8	2	70	0.7693	1.7950	2.5642	AOE47672.1	NO
LSDV103	A12L	M101L	Putative virion core protein	20,923	5	7	2	94	0.4779	0.3824	0.8603	AOE47679.1	NO
LSDV109	A17L	M107L	Putative phosphorylated IMV membrane protein	22,184	11	11	1	5	25.6040	28.9398	54.5438	AOE47685.1	NO
LSDV110	A18R	M108R	Putative DNA helicase transcriptional elongation factor	56,480	12	15	1	91	0.6197	0.2833	0.9030	AOE47686.1	NO
LSDV116	A24R	M114R	RNA polymerase subunit	132,628	58	70	2	34	2.4203	3.2346	5.6549	AOE47692.1	NO
LSDV120	A30L	M118L	IMV membrane protein	8,576	3	4	0	90	0.1166	0.8162	0.9328	AOE47696.1	NO
LSDV122	A33R	M121R	EEV glycoprotein	22,578	7	9	1	36	2.6132	2.9232	5.5364	AOE47698.1	NO
LSDV123	A34R	M122R	IEV and EEV membrane glycoprotein	19,499	10	11	2	23	2.9745	3.6925	6.6670	AOE47699.1	NO
LSDV128	A38L	M128L	CD47-like protein	35,581	4	5	2	92	0.5059	0.3654	0.8713	AOE47704.1	NO
LSDV129		M130R	Hypothetical protein	14,138	4	4	1	99	0.3537	0.2829	0.6366	AOE47705.1	NO
LSDV134		M134R	LD134	230,357	76	110	1	59	1.8754	1.4022	3.2775	AAN02702.1	NO
LSDV135	B19R	M135R	Putative IFN-alpha/beta binding protein	42,105	11	13	1	97	0.3800	0.3563	0.7363	AOE47710.1	NO
LSDV141	B5R	M144R	EEV host range protein	26,148	12	13	1	46	2.2181	2.1417	4.3598	AOE47716.1	NO
LSDV142	N1L	M146R	Putative secreted virulence factor	15,792	5	6	2	78	0.5699	1.2665	1.8364	AOE47717.1	NO
LSDV143		M147R	Tyrosine protein kinase-like protein	35,544	5	7	2	95	0.2532	0.5908	0.8440	AOE47718.1	NO
LSDV146	K4L	M022L	Phospholipase-D-like protein	47,850	21	24	2	17	4.2006	5.6844	9.8851	AOE47721.1	NO
LSDV147	B4R	M149R	Ankyrin repeat protein	58,625	7	10	2	105	0.2729	0.2900	0.5629	AOE47722.1	NO
LSDV149	C12L	M151R	Serpin-like protein	38,873	5	7	2	98	0.2058	0.4630	0.6688	AOE47724.1	NO
LSDV150	A52R	M139R	Hypothetical protein	19,255	6	7	2	71	0.8310	1.7138	2.5448	AOE47725.1	NO

^*a*^ Best matching ORF from the vaccinia virus genome as previously described (27, 28).

^*b*^ Best matching ORF from the myxoma virus genome as previously described (27, 28).

^*c*^ Accession numbers are from the Genbank or Uniprot database.

^*d*^ Data in Table 1 are compiled from five different LSDV preparations per condition with two conditions (tartrate and sucrose continuous gradients). Only proteins with at least two peptides were counted. ORF-VACV: predicted orthologous gene in vaccinia virus for each considered LSDV gene. ORF-MYXV: predicted orthologous gene in myxoma virus for each considered LSDV gene. Number of hits across replicates: indicates how many times (out of five replicates) the protein was detected within the defined limits (i.e., considered as packaged).

**Fig 3:**
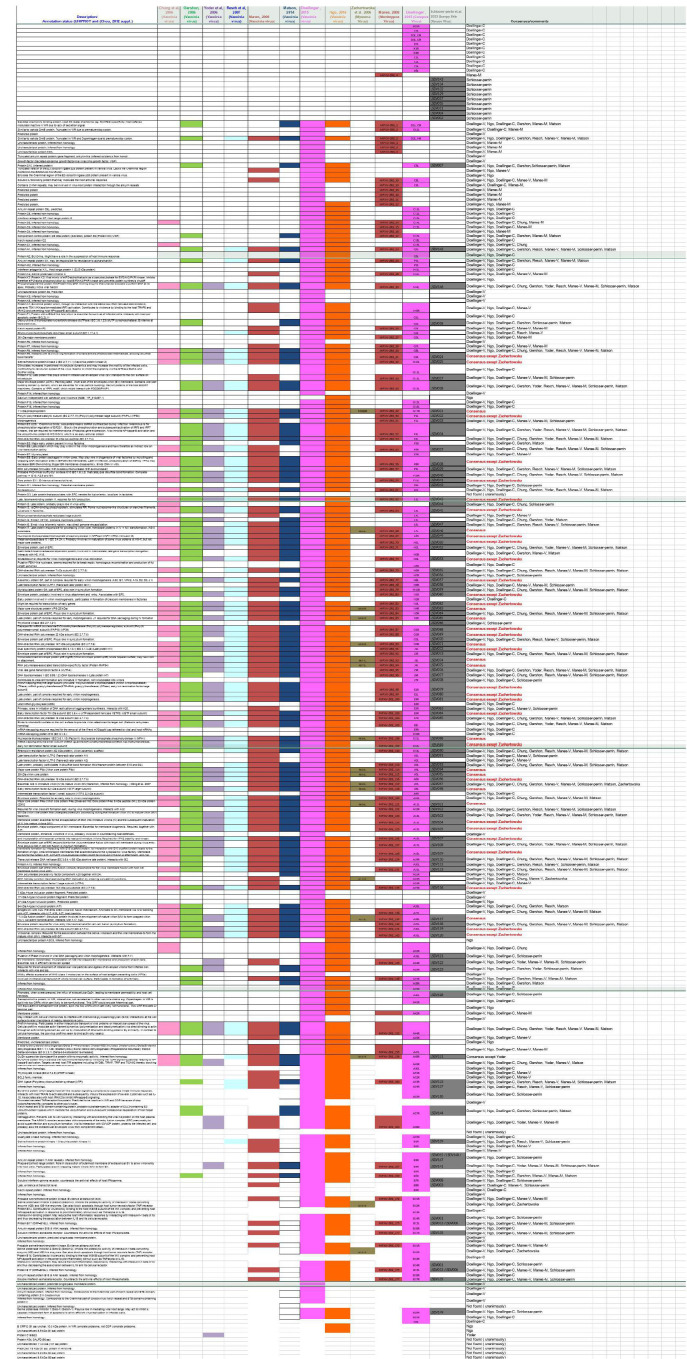
Proteome of vaccinia virus, monkeypox virus, cowpox virus, myxoma virus, and lumpy skin disease virus. Visual impression based on Table S2 in the supplemental material. Proteins detected in the different proteomics studies are represented in the various columns. Studies are ordered as follows: from left to right, the oldest study [Chung et al. (56)] on vaccinia to the most recent one [Ngo et al. (40)] followed by three non-vaccinia-based studies ordered again chronologically [respectively, myxoma virus (61), monkeypox (58), and cowpox (57)]. Colors indicate the studies as follows: the results of the current study are shown in gray. Other colors indicate previous studies, either published [red for Chung et al. (56), mauve for Yoder et al. (60), blue for Resch et al. (59), brown for Manes et al. (58), dark blue for Matson et al. (43), pink for Doellinger et al. (57), orange for Ngo et al. (40), and greenish yellow for Zachertowska et al. (61)] or unpublished [green for the Gershon laboratory study from Ngo et al. (40)]. The table is taken directly from reference (40) with permission and supplemented with the results of proteomic studies carried out on viruses other than vaccinia virus.

Figure 3 (Table S2 in the supplemental material) provides a detailed comparison of the 111 detected LSDV proteins with the viral proteins detected in the analyses of virion preparations using a similar shotgun proteomics approach on orthopoxviruses [VACV, Cowpox virus (CPXV), and Monkeypox virus (MPXV)] (40, 43, 56 – 60) and leporipoxviruses (MYXV) (61). Excluding leporipoxvirus for which deficiencies in MS instrumentation performance were reported (61), the comparison of the proteome of the LSDV preparation with the proteomes of orthopoxvirus preparations revealed a set of 43 homologous viral proteins universally detected in all the virion preparations analyzed (Fig. 3; Table S2 in the supplemental material). In addition, nine LSDV proteins (LSDV002, LSDV004, LSDV011, LSDV015, LSD017, LSDV032, LSDV129, LSDV134, and LSDV143), for which no counterpart was identified in the VACV genome (26, 27), were detected in the LSDV preparation (Fig. 3; Table S2 in the supplemental material).

**Fig 4:**
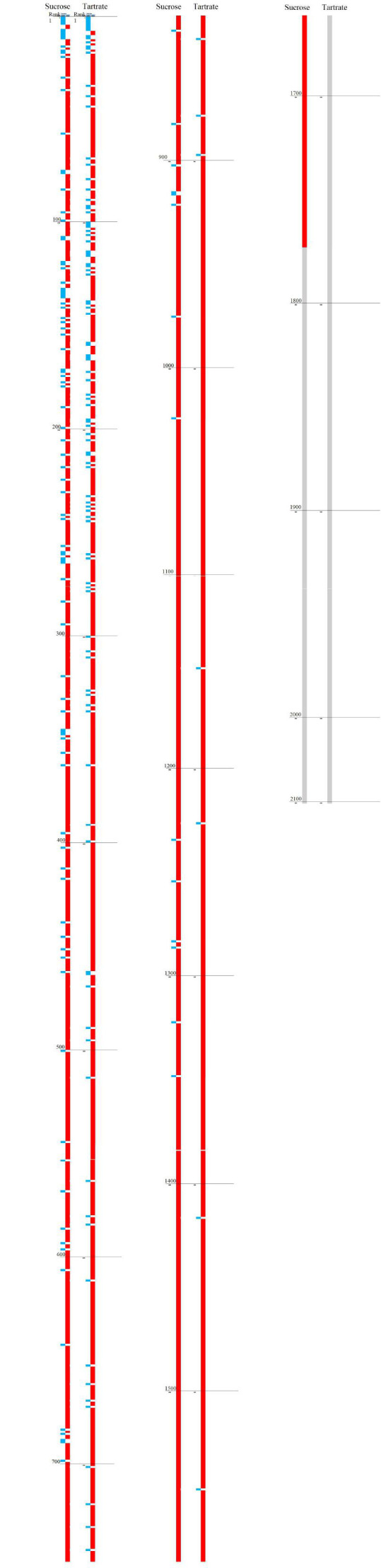
Relative abundance (NSAF) of lumpy skin disease and host proteins identified in our viral preparations (sucrose and tartrate). Visual impression based on Table S3 in the supplemental material. On the left, proteins identified in sucrose gradient-purified virus preparation. On the right, proteins identified in tartrate gradient-purified virus preparation. Proteins were ranked by descending NSAF score (the highest protein on the *y*-axis being the protein with the highest score and therefore the highest relative abundance). For each condition (sucrose and tartrate), ranking numbers were split into two groups, namely “LUMPY” (blue for lumpy skin disease virus proteins) and “BOS” (red for bovine proteins). Proteins which could not be certified (less than two spectra detected) are shown in gray.

A total of 10,573 specific MS/MS spectra made it possible to identify 1,473 bovine proteins, certified with high confidence. Of at least 22,000 *in silico* predicted protein coding sequences (CD29S) annotated on the *Bos taurus* genome (62), 1,473 bovine proteins were identified at least once across all replicates. We noted an average of 7.2 specific peptides for each protein excluding the proteins with one single specific spectrum with a range of 2 to 100 specific peptides. The peptide coverage varies from 1% to 93% with the best covered proteins being seven proteins with 80%, 82%, 84%, 86%, 88%, 89%, and 93% coverage and 56, 47, 27, 6, 46, 25, and 15 specific spectra attributed to them, respectively. The number of host proteins exceeds the number of viral proteins by a factor of 12 (sucrose) and 11 (tartrate) (Table S1) due to the high sensitivity of the tandem mass spectrometer used. Similar results [number of host proteins and ratio (number host proteins/number of viral proteins)] were reported in the most recent shotgun proteomic analyses of orthopoxvirus preparations (40, 57, 58). Additional information is provided in Fig. 4 (Table S3 in the supplemental material) showing the respective relative abundances (NSAF) observed for the viral proteins compared to those of the host cell proteins in the current study. In the LSDV preparation, the detected host proteins had predominantly lower ranking numbers than the detected virus proteins, as previously reported in the study by Ngo et al. (40).

These results taken together highlight the need to distinguish the proteome of the virion preparation from the proteome of the virion itself.

### Which proteins are packaged?

Considering the 111 viral proteins detected (Table 1) in the LSDV preparation, of the 156 possible hypothetical ORFs of the LSDV genome, it seemed that not all of them are packaged “on purpose” in the infectious viral particles and that numerous contaminants, of host and viral origin, may have co-purified despite efforts made in the purification processes. In line with observations from Ngo et al. (40), low abundance packaged proteins may overlap in abundance with medium to low abundance contaminants. Analysis of the relative abundance distributions of viral and cellular proteins (Fig. 4; Table S3 in the supplemental material) emphasizes the difficulty of using rank as the only parameter to sort packaged proteins from contaminating proteins. Indeed, the simple ranking through NSAF did not appear sufficient to stipulate whether or not proteins are actively packaged in virion particles. There was a need to provide further insight into the selective protein packaging. With this in mind and in line with the Ngo et al. study (40), we designed our virus purification protocol, taking advantage of two types of centrifugal separations, relying on distinct density gradient media, namely a rate-zonal (or nonequilibrium) separation in a continuous sucrose gradient (sucrose) and an isopycnic (or equilibrium buoyant density) separation in a continuous potassium tartrate gradient. Therefore, unlike packaged proteins, which would show a proportional distribution between the two ultracentrifugation conditions, unpackaged proteins would preferentially contaminate one condition or the other and exhibit a skewed distribution toward either condition. Under our protocol, it was therefore expected that we would observe two different distribution patterns between the two ultracentrifugation conditions: a skewed distribution for contaminants alongside proportional distribution for packaged proteins. In practice, under our protocol, the distribution pattern of a given protein, skewed or proportional toward one condition or the other, seems to be reasonably approached by calculating the tartrate/sucrose NSAF ratio (*Q* ratio).

In order to address the question about which protein is packaged or not, we proceeded in three steps.

**Fig 5:**
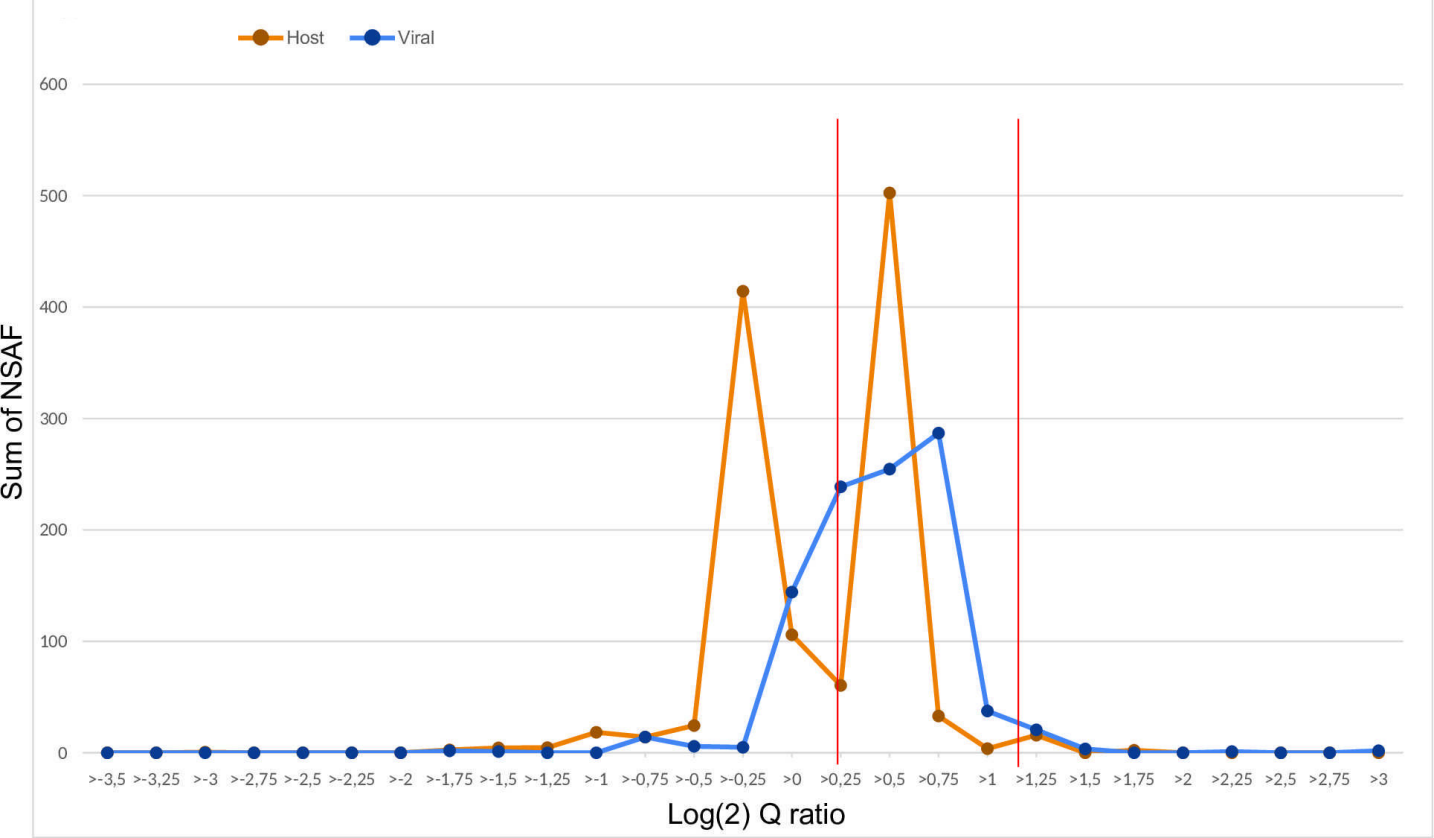
Selection of the “packaged region.” Line histogram showing relative quantification of proteins present in sucrose versus tartrate purified LSD virion preparations based on label-free quantification. Only proteins quantitated with multiple peptides are shown. LSDV proteins are shown in blue and host proteins in orange. The log_2_ quantitation ratio (*Q* ratio), shown on the *x*-axis, is obtained by dividing the sum of the tartrate NSAF score across all replicates by the sum of the sucrose NSAF score across all replicates. This was implemented for each protein (viral and host) comprising our viral preparations, and the ratios obtained for each protein were placed along Fig. 5’s *x*-axis, defining contiguous tartrate/sucrose ratio class intervals (bins). For example, “>0.5” corresponds to a tartrate/sucrose ratio from 2^0.5^ to 2^0.75^ (log_2_ scale). Considering thereafter every protein for which the *Q* ratio falls within the same bin on the *x*-axis, the NSAF of both virion purification conditions (sucrose and tartrate) were then summed up to build Fig. 5’s y-axis. Proteins in bins falling between the red vertical lines (“packaged region”) were considered packaged.

First, the tartrate/sucrose NSAF (*Q* ratio) was used to achieve an overview on the distribution pattern of the whole set of detected proteins according to the *Q* ratio. Since our experimental setting used five replicates per condition, we have summed the NSAF corresponding to each of the replicates of the tartrate condition and divided this total by the sum of the NSAF corresponding to each of the replicates of the sucrose condition. This was implemented for each protein (viral and host) comprising our purified viral extracts and the ratios obtained for each protein were placed along Fig. 5’s *x*-axis, defining contiguous tartrate/sucrose ratio class intervals (bins). Considering thereafter every protein, for which the *Q* ratio falls within the same bin on the *x*-axis, the NSAF of both conditions (sucrose and tartrate) was then summed up to build Fig. 5’s *y*-axis. Applying this procedure to all the identified proteins, we obtained an overview on the distribution of viral (blue line) and host proteins (orange) represented in Fig. 5. A major viral peak comprises almost all viral proteins with a tartrate/sucrose ratio bin located between >−0.25 and >1 (log_2_ scale). Two host protein peaks are also clearly identified; one is clearly separated from the viral protein peak (tartrate/sucrose ratio bin below 0 on the log_2_ scale) while the other overlaps the viral protein peak (tartrate/sucrose ratio bin between >0.25 and >0.75 on the log_2_ scale).

Second, once we had this overview on the distribution pattern of the whole set of detected proteins according to the *Q* ratio, it was then possible, in line with Ngo et al. (40), to try to define bin limits separating actively packaged proteins from likely contaminant proteins. We considered that bin limits, ranging from >0 to >1 (log_2_ scale), would correspond to a proportional distribution pattern for a given protein between the two ultracentrifugation conditions. As illustrated in Fig. 5, these limits (vertical red lines) include almost all of the viral protein peak as well as one of the two host proteins peak.

Third, once these limits were defined, it was then possible to analyze individually each protein, characterizing its tartrate/sucrose NSAF values for each of the five replicates and assessing the number of replicates for which these values were within/outside the defined limits (hereinafter referred to as “packaged region”). As a selection criterion, we considered that a protein demonstrating a proportional and stable distribution between the two conditions would display values inside the packaged region for at least three out of five replicates. Conversely, proteins with at least three values outside the packaged region would be considered as a contaminant protein exhibiting a skewed affinity toward one condition or the other.

In conclusion, following the procedure described above, protein analysis was first undertaken for the 111 viral proteins and resulted in the selection of 66 proteins (59%) which were considered, under our standards, as candidates for packaging into MV virion. Of these 66 proteins, we found nine that fell within the packaged region throughout all five replicates (LSDV 18, 28, 38, 43, 45, 59, 72, 94, and 97). In addition, 18 proteins were stably detected within the packaged region in four out of five replicates (LSDV 1, 17, 37, 41, 42, 50, 53, 58, 63, 70, 73, 83, 91, 104, 113, 132, 137, and 139). Furthermore, we found 39 proteins falling within the packaged region in three out of five replicates (LSDV 3, 4, 7, 12, 13, 31, 40, 44, 52, 57, 60, 64, 65, 69, 71, 74, 79, 80, 81, 84, 89, 90, 93, 95, 98, 101, 102, 105, 107, 108, 111, 117, 118, 119, 121, 131, 133, 144, and 148).

**Fig 6:**
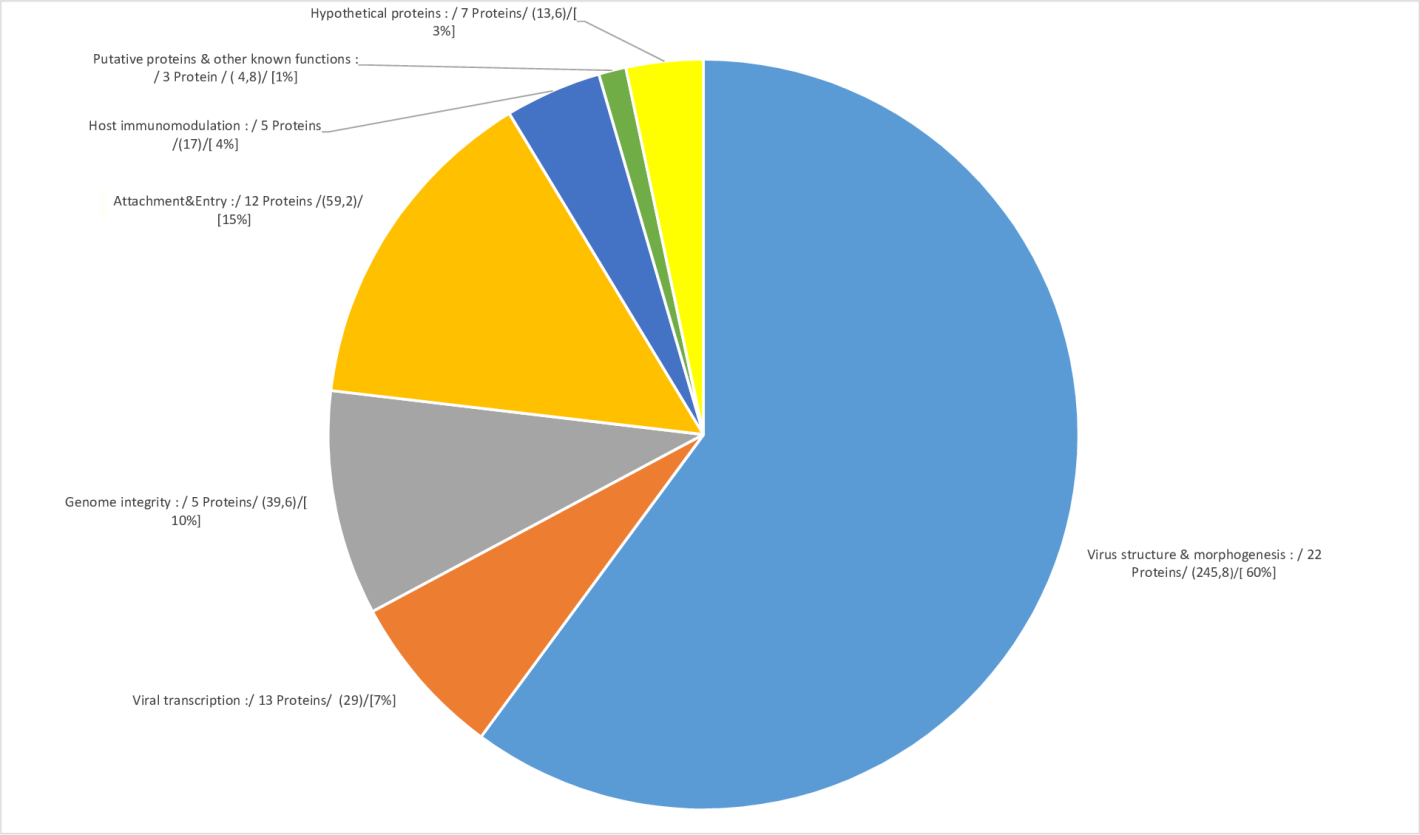
Functional characterization of selected MV proteins of purified LSDV preparations. Selected LSDV proteins can be distributed according to functional groups, and the number of proteins classified in each group is shown in the figure. For each protein, an NSAF protein value (NSAF_p_) was calculated by adding the mean of the NSAF values of the five replicates of the tartrate condition to the mean of the NSAF values of the five replicates of the sucrose condition. This was implemented for each of the 66 selected viral protein. The total NSAF value of the virus preparation corresponding to the 66 selected LSDV proteins (NSAF_vir.prep_.) was obtained by adding the NSAF_p_ of the 66 selected LSDV proteins. The total NSAF value of a given functional group (NSAF_f.group_) was obtained by summing the NSAF_p_ of the LSDV proteins grouped within this functional group. For each functional group, the value of the NSAF_f.group_ is shown in parentheses in the figure. In addition, for each functional group, the percentage of NSAF_f.group_ relative to the NSAF_vir.prep_. is calculated and indicated in square brackets in the figure. The size of the pie chart portion associated with each functional group is determined on the basis of this percentage. Information on the proteins grouped in each functional group is reported below. Some 22 proteins (60% of the total NSAF of viral proteins) may be identified as maintaining viral structure through the membrane or viral core (LSDV 28, 31, 37, 38, 40, 41, 50, 53, 57, 63, 65, 80, 81, 90, 94, 95, 97, 101, 102, 104, 105, and 107,). Thirteen proteins (7% of the total NSAF of viral proteins) were involved in viral transcription, most with early transcription functions but also intermediate and late transcription functions too (LSDV 3, 58, 69, 71, 79, 84, 89, 91, 98, 111, 119, 131, and 139). Five proteins (10% of the total NSAF of viral proteins) were associated with genome integrity with DNA folding and packing, and with DNA reparation (LSDV 43, 45, 83, 121, and 133). Twelve proteins (15% of the total NSAF of viral proteins) were identified for virus entry in host cells and virus attachment regulation to avoid superinfection (LSDV 44, 52, 59, 60, 64, 70, 73, 74, 108, 113, 117, and 118), and five proteins (4% of the total NSAF of viral proteins) were identified as potential immunomodulatory effectors (LSDV 1, 7, 13, 72, and 144). Finally, three proteins (1% of the total NSAF of viral proteins) may be grouped within putative proteins/other (LSDV 12, 18, and 148) and seven proteins (2.6% of the total NSAF of viral proteins) within hypothetical proteins with no postulated function per homology with vaccine (LSDV 4, 7, 17, 42, 93, 132, and 137).

Based upon previously predicted functions (26, 27), the 66 selected viral proteins, candidates for packaging, were divided between seven functional categories (Fig. 6).

According to such grouping, 22 proteins (60% of the total NSAF of viral proteins) may be identified as maintaining viral structure through the membrane or viral core (LSDV 28, 31, 37, 38, 40, 41, 50, 53, 57, 63, 65, 80, 81, 90, 94, 95, 97, 101, 102, 104, 105, and 107).

Thirteen proteins (7% of the total NSAF of viral proteins) were involved in viral transcription, most with early transcription functions but also with intermediate and late transcription functions too (LSDV 3, 58, 69, 71, 79, 84, 89, 91, 98, 111, 119, 131, and 139).

Five proteins (10% of the total NSAF of viral proteins) were associated with genome integrity with DNA folding and packing, and with DNA reparation (LSDV 43, 45, 83, 121, and 133).

Twelve proteins (15% of the total NSAF of viral proteins) were identified for virus entry in host cells and virus attachment regulation (LSDV 44, 52, 59, 60, 64, 70, 73, 74, 108, 113, 117, and 118), and five proteins (4% of the total NSAF of viral proteins) were identified as potential immunomodulatory effectors (LSDV 1, 7, 13, 72 and 144). Finally, three proteins (1% of the total NSAF of viral proteins) may be grouped within putative proteins/others (LSDV 12, 18, and 148) and seven proteins (2.6% of the total NSAF of viral proteins) within hypothetical proteins with no postulated function per homology with vaccine (LSDV 4, 7, 17, 42, 93, 132, and 137). While these latter proteins were hitherto annotated as hypothetical, their identification through tandem MS certified their existence, and they should no longer be considered hypothetical.

The LSDV proteins, selected in this study as candidates for packaging and classified into functional groups, were then analyzed against a list of well-characterized proteins for which there are consistent data demonstrating their incorporation into the viral particle (39). These non-omics data were produced in studies using fundamentally distinct approaches (39). In addition, because the selection method of the LSDV proteins developed in the present study is a derivative of that developed by Ngo et al. (40), it was considered relevant to also include the proteins selected by Ngo et al. in this comparative analysis.

This comparative analysis was first performed on 29 viral proteins listed by Condit et al. that can be grouped into the category of viral structure and morphogenesis (Table 2) (39). Of these 29 viral proteins: (i) fifteen proteins (A3, A4, A13, A14, A15, D2, D3, E8, E10, E11, F17, G1, G7, L4, J1, and LSDV counterparts) were jointly selected in the current study and by Ngo et al. (40), (ii) four proteins (A10, A11, D13, G4, and LSDV counterparts) were only selected in the current study, (iii) six proteins (A12, A17, A30, F10, I5, I7, and LSDV counterparts) were only selected by Ngo et al., (iv) three proteins (A9, A14.5, A22, and LSDV counterparts) were neither selected in the current study nor by Ngo et al., and (v) one protein without LSDV counterpart was selected by Ngo et al. (A26) (Table 2). Still in that same protein group, although not listed by Condit et al., two proteins (A6, E6, and LSDV counterparts) were nonetheless selected jointly in this current study and by Ngo et al., one protein (LSDV028/F13 orthologs) was selected only in the current study, two proteins (F12, E2, and LSDV counterparts) were selected only by Ngo et al., and one protein without LSDV counterpart (A25) was selected only by Ngo et al.

**TABLE 2:** LSDV proteins selected as candidates for packaging^*a*^

Locus tag	Gene name (WR)	Condit et al. 2006 (39)	Ngo et al. 2016 (40)	Schlosser et al., LSDV ORF equivalents
Virus structure and morphogenesis				
VACWR049	F10L	**C^*c*^**	**S^*d*^**	LSDV025; **D^*e*^**
VACWR051	F12L		**S**	LSDV027; **D**
VACWR052	F13L		**D**	LSDV028; **S**
VACWR056	F17L	**C**	**S**	LSDV031; **S**
VACWR058	E2L		**S**	LSDV033; **D**
VACWR062	E6R		**S**	LSDV037; **S**
VACWR064	E8R	**C**	**S**	LSDV038; **S**
VACWR066	E10R	**C**	**S**	LSDV040; **S**
VACWR067	E11L	**C**	**S**	LSDV041; **S**
VACWR074	I5L	**C**	**S**	LSDV046; **ND^*f*^**
VACWR076	I7L	**C**	**S**	LSDV048; **D**
VACWR078	G1L	**C**	**S**	LSDV050; **S**
VACWR081	G4L	**C**	**D**	LSDV053; **S**
VACWR085	G7L	**C**	**S**	LSDV057; **S**
VACWR091	L4R	**C**	**S**	LSDV063; **S**
VACWR093	J1R	**C**	**S**	LSDV065; **S**
VACWR107	D2L	**C**	**S**	LSDV080; **S**
VACWR108	D3R	**C**	**S**	LSDV081; **S**
VACWR118	D13L	**C**	**D**	LSDV090; **S**
VACWR122	A3L	**C**	**S**	LSDV094; **S**
VACWR123	A4L	**C**	**S**	LSDV095; **S**
VACWR125	A6L		**S**	LSDV097; **S**
VACWR128	A9L	**C**	**D**	LSDV100; **ND**
VACWR129	A10L	**C**	**D**	LSDV101; **S**
VACWR130	A11R	**C**	**D**	LSDV102; **S**
VACWR131	A12L	**C**	**S**	LSDV103; **D**
VACWR132	A13L	**C**	**S**	LSDV104; **S**
VACWR133	A14L	**C**	**S**	LSDV105; **S**
VACWR134	A14.5L	**C**	**ND**	LSDV106; **ND**
VACWR135	A15L	**C**	**S**	LSDV107; **S**
VACWR137	A17L	**C**	**S**	LSDV109; **D**
VACWR142	A22R	**C**	**D**	LSDV114; **ND**
VACWR148	A25		**S**	**No ORF^*g*^**; **ND**
VACWR149	A26L	**C**	**S**	**No ORF**; **ND**
VACWR153	A30L	**C**	**S**	LSDV120; **D**
Viral transcription				
VACWR035	K4L	**C**	**S**	LSDV146; **D**
VACWR047	F8L		**S**	**No ORF**; **ND**
VACWR057	E1L	**C**	**S**	LSDV032; **D**
VACWR060	E4L	**C**	**S**	LSDV035; **ND**
VACWR069	O2L	**C**	**S**	**No ORF**; **ND**
VACWR077	I8R	**C**	**S**	LSDV049; **D**
VACWR083	G5.5R	**C**	**S**	LSDV055; **D**
VACWR086	G8R		**D**	LSDV058; **S**
VACWR090	L3L	**C**	**S**	LSDV062; **D**
VACWR095	J3R	**C**	**S**	LSDV068; **D**
VACWR096	J4R	**C**	**S**	LSDV069; **S**
VACWR098	J6R	**C**	**S**	LSDV071; **S**
VACWR102	H4L	**C**	**S**	LSDV075; **D**
VACWR103	H5R	**C**	**S**	LSDV076; **D**
VACWR104	H6R	**C**	**S**	LSDV077; **D**
VACWR106	D1R	**C**	**S**	LSDV079; **S**
VACWR111	D6R	**C**	**S**	LSDV084; **S**
VACWR112	D7R	**C**	**S**	LSDV085; **D**
VACWR116	D11L	**C**	**S**	LSDV088; **D**
VACWR117	D12L	**C**	**S**	LSDV089; **S**
VACWR119	A1L		**S**	LSDV091; **S**
VACWR121	A2.5L	**C**	**S**	LSDV093; **S**
VACWR124	A5R	**C**	**S**	LSDV096; **D**
VACWR126	A7L	**C**	**S**	LSDV098; **S**
VACWR138	A18R	**C**	**S**	LSDV110; **D**
VACWR139	A19L		**D**	LSDV111; **S**
VACWR144	A24R	**C**	**S**	LSDV116; **D**
VACWR152	A29L	**C**	**S**	LSDV119; **S**
VACWR171	A45R	**C**	**S**	LSDV131; **S**
VACWR183	B1R	**C**	**S**	LSDV139; **S**
VACWR191	B9R		**ND**	LSDV003; **S**
Genome integrity				
VACWR070	I1L	**C**	**S**	LSDV043; **S**
VACWR072	I3L		**S**	LSDV045; **S**
VACWR075	I6L	**C**	**S**	LSDV047; **D**
VACWR110	D5R		**D**	LSDV083; **S**
VACWR155	A32L	**C**	**D**	LSDV121; **S**
VACWR176	A50R		**S**	LSDV133; **S**
Attachment and entry				
VACWR048	F9L^*b*^		**S**	LSDV024; **D**
VACWR069.5	O3L^*b*^		**ND**	**No ORF**; **ND**
VACWR071	I2L	**C**	**D**	LSDV044; **S**
VACWR079	G3L^*b*^	**C**	**S**	LSDV052; **S**
VACWR087	G9R^*b*^	**C**	**S**	LSDV059; **S**
VACWR088	L1R^*b*^	**C**	**S**	LSDV060; **S**
VACWR092	L5R^*b*^	**C**	**S**	LSDV064; **S**
VACWR097	J5L^*b*^	**C**	**D**	LSDV070; **S**
VACWR100	H2R^*b*^	**C**	**D**	LSDV073; **S**
VACWR101	H3L	**C**	**S**	LSDV074; **S**
VACWR113	D8L	**C**	**S**	**No ORF**; **ND**
VACWR136	A16L^*b*^	**C**	**S**	LSDV108; **S**
VACWR140	A21L^*b*^	**C**	**S**	LSDV113; **S**
VACWR150	A27L	**C**	**S**	LSDV117; **S**
VACWR151	A28L^*b*^	**C**	**S**	LSDV118; **S**
Host immunomodulation				
VACWR010, VACWR209	C10L		**D**	LSDV007; **S**
VACWR099	H1L	**C**	**S**	LSDV072; **S**
VACWR180	A55R		**D**	LSDV144; **S**
VACWR197	B15R		**D**	LSDV001; **S**
VACWR198	B16L		**D**	LSDV013; **S**

^*a*^ This table represents (i) VACV proteins selected as virion proteins by converging non-omics studies [Condit et al. (39)] (third column from the left), (ii) VACV proteins selected as candidates for packaging [Ngo et al. (40)] (fourth column from the left), and (iii) LSDV proteins selected as candidates for packaging in the current study (last column). For the sake of clarity, these selected proteins have been distributed according to functional groups as follows: virus structure and morphogenesis, viral transcription, genome integrity, entry and attachment, and host immunomodulation.

^*b*^ Entry fusion complex.

^*c*^ C, converging studies.

^*d*^ S, selected.

^*e*^ D, detected.

^*f*^ ND, non-detected.

^*g*^ NO ORF, ORF not identified in LSDV genome.

The comparative analysis was then performed on three viral proteins listed by Condit et al. (39) that can be grouped into the category of genome integrity proteins (Table 2). Of these three proteins: (i) one protein (I1 and its LSDV counterpart) was jointly selected in the current study and by Ngo et al. (40), (ii) one protein (A32 and its LSD counterpart) was only selected in the current study, and (iii) one protein (I6 and its LSD counterpart) was only selected by Ngo et al. Still in the same viral protein group, although not listed by Condit et al., two proteins (A50, I3, and LSDV counterparts) were selected jointly in the current study and by Ngo et al., and one protein (D5 and its LSD counterpart) was only selected in the current study.

Next, the comparative analysis was performed on 13 viral proteins listed by Condit et al. (39) that can be grouped into the category of attachment and entry proteins (Table 2). Of these 13 proteins: (i) nine proteins (A16, A21, A27, A28, G3, G9, H3, L1, L5, and LSDV counterparts) were jointly selected in the current study and by Ngo et al., (ii) three proteins (H2, I2, J5, and LSDV counterparts) were only selected in the current study, and (iii) one protein without an LSDV counterpart (D8) was only selected by Ngo et al. Still in the same viral protein group, although not listed by Condit et al., one viral protein (F9L) was only selected by Ngo et al.

The comparative analysis was then performed on 26 proteins listed by Condit et al. (39) that can be grouped into the category of viral transcription proteins (Table 2). Of these 26 proteins: (i) 10 proteins (A2.5, A7, A29, A45, B1, D1, D6, D12, J4, J6, and LSDV counterparts) were jointly selected in the current study and by Ngo et al., (ii) 15 proteins (A5, A18, A24, D7, D11, E1, E4, G5.5, H4, H5, H6, I8, J3, K4, L3, and LSDV counterparts) were only selected by Ngo et al., and (iii) 1 protein without an LSDV counterpart (O_2_) was only selected by Ngo et al. Still in the same viral protein group, although not listed by Condit et al., (i) one protein (A1 and its LSDV counterpart) was jointly selected in the current study and by Ngo et al., (ii) three proteins were only selected in the current study (A19, B9, G8, and LSDV counterparts), and (iii) one protein without an LSDV counterpart (F8) was only selected by Ngo et al.

Finally, the comparative analysis was performed on the unique viral protein (H1) listed by Condit et al. (39), which can be classified into the category of immunomodulatory proteins (Table 2). In this group, H1 was jointly selected in the current study and by Ngo et al. Still in the same viral protein group of immunomodulatory effectors, although not listed by Condit et al., four proteins (A55, B15, B16, C10, and LSDV counterparts) were only selected in the current study.

After applying our selection method to all detected LSDV proteins, this method was applied to the 1,473 bovine proteins detected in our viral preparation. Table S4 in the supplemental material shows a total of 65 proteins always found in the packaged region of the quantitation histogram in the five experiments. Among this set of 65 bovine proteins, whereas some proteins were previously found to be associated to virus particles (56, 63, 64), several of these selected host proteins belong to protein classes that have been previously reported to be associated with low-level contaminations of MV preparations, including cytoskeletal proteins, chaperones, and mitochondrial proteins as well as proteins involved in vesicular transport/protein trafficking (RABs) located in a variety of membrane compartments in the uninfected cell (40, 59, 65).

## DISCUSSION

This study aimed to characterize for the first time the list of viral proteins incorporated into the infectious LSDV particle. To initiate this characterization work, LSDV KSGP-0240 was chosen due to its unique genetic and biological features. MV particles were purified from MDBK cells infected with the KSGP-0240 strain through a multistep ultracentrifugation workflow including either rate-zonal centrifugation in a continuous sucrose gradient or isopycnic centrifugation in a continuous tartrate gradient. The purified viral fractions were then analyzed using MS, and a total of 111 viral proteins and 1,473 cellular proteins were identified. In order to discriminate packaged proteins from contaminant proteins, a specific analytical methodology was developed, taking advantage of the differential properties of the two-density gradient media (tartrate and sucrose). Applying our methodology to the total number of viral proteins detected in our purified viral preparations, we finally concluded that 66 viral proteins are candidates for packaged viral proteins.

While Vandenbussche et al. (27) annotating the KSGP-0240 strain genome, theoretically predicted 156 ORFs, our study actually detected 111 proteins, demonstrating for the first time their synthesis during the viral infection course. Regarding the total number of proteins detected, it is important to stress that our study especially targeted MV infectious particles, differing in particular from studies addressing the viral infectome (66). The total number of viral proteins detected in our study reaches >71% of the total number of theoretically predicted ORFs comprising the genome of the KSGP-0240 strain (26, 27). Ten ORFs, which have been annotated publicly as “putative” or “hypothetical,” but for which no expression had been demonstrated, are now confirmed through the detection of their protein product. The genome coverage, corresponding to the 111 LSDV proteins detected in this study (number of proteins detected in virus preparations out of the total number of predicted ORFs in the whole genome) is in line with the progression of reported genome coverages in poxvirus proteomics studies performed over recent decades (Fig. 3; Table S2 in the supplemental material), ranging from 10% (61) to 82% (40). A critical parameter that has decisively contributed to this evolution is the progressive improvement in the performance of MS instruments, which has resulted in a hitherto comprehensive description of the proteome of viral preparations. Regarding the increase in the genome coverages achieved over recent decades, it is also worth mentioning the key role played in the present study by the setting of the parameters (exclusion time for MS/MS acquisition and activation threshold) in maximizing the number of peptides to monitor and thereby in identifying low-abundance proteins (54, 55).

Poxviral studies, performed on MV virions purified through centrifugation gradients (40, 56, 60, 65, 67, 68), provided a previous warning against the risk of contaminants and insisted on the absolute requirement of differentiating between a low level of specific packaging and non-specific packaging or contamination. The contamination issue was addressed in our study in line with a previous study performed on VACV (40).

Basically, using two methods of purification that rely on different density gradient media (tartrate and sucrose) with distinct affinity toward contaminant proteins would result in observing two distribution patterns between the two conditions. Contrary to packaged proteins, which would show proportionality between the two conditions, non-packaged proteins would preferentially contaminate one condition or the other. Based upon this assumption, we developed a methodology similar to the one previously proposed by Ngo et al. for VACV MV (40). Applying this methodology to the 111 viral proteins initially detected under our experimental settings, we selected only 66 viral proteins which, in our defined standards, could be considered as candidate packaged viral proteins. These 66 LSDV proteins and their corresponding ORFs represent 42% of the total number of ORFs comprising the LSDV full genome, which approximates the percentage achieved for VACV MV proteins selected by Ngo et al. (34%) (40). However, it is clear that our current effort to exclude contaminants from the specific packaged virion proteins must be pursued, including complementary experimental approaches and especially those relying upon alternate purification methods using density gradient media differentially prone to protein contamination. So, using successively continuous gradients with density gradient media other than the one used in this study (CsCl, iodixanol, nycodenz) (57, 65, 69, 70) could extend our results and bring additional relevant information regarding the contamination issue. Besides, including at the end of the workflow an additional purification step on a chromatographic column could make it possible to achieve an even higher purity index and reduce the protein contamination load (71).

To characterize the functions of the detected proteins of our viral purified extracts, we had to rely on previous studies (26, 27), which undertook an in-depth annotation of the LSDV whole genome and proposed theoretical ORFs, corresponding proteins and their postulated function. Based upon these predicted functions, the 66 selected viral proteins, candidates for packaging, were divided into seven functional categories (Fig. 6). These 66 LSDV proteins were then analyzed against (i) a list of well-characterized proteins for which there are consistent data demonstrating their incorporation into the viral particle (39) and (ii) a list of proteins selected by Ngo et al. (40) using a selection method directly related to the selection method used in the current study (Table 2).

Among our protein data set, the attachment and entry group is especially remarkable with 12 proteins (92%) of the 13 VACV proteins previously identified as constitutive of the VACV MV virion (39), which have their LSDV counterparts selected as constitutive of the LSDV MV virion (Table 2). Of these 13 VACV proteins, Ngo et al. (40) selected 10 proteins (Table 2). It is noteworthy that two proteins, F9 and O3, constitutive of the entry fusion complex (EFC) together with A16, A21, A28, G3, G9, H2, J5, L1, and L5 (3), were not included among the proteins constitutive of the VACV MV virion listed by Condit et al. (39). All the nine EFC proteins of this entry/attachment group, listed by Condit et al. (39), had their LSDV orthologs selected in the current study (Table 2). The two EFC proteins missing from the EFC proteins are either not selected but detected (LSDV024/F9 ortholog) or undetected (O3). Selecting these nine LSDV orthologs as candidate-packaged viral proteins may advocate in favor of the conservation, throughout the POXV family, of an EFC embedded in the membrane of MV particle. The lack of detection of O3 among the EFC components constitutive of the LSDV MV particle is expected since this VACV protein is predicted not to have any ortholog in the LSDV genome (26, 27). The O3 protein is neither detected in the study by Ngo et al. (Table 2) nor detected in any of the poxvirus proteomics studies (Fig. 3; Table S2 in the supplemental material). Since it has been recently demonstrated that the small hydrophobic VACV O3 protein seems to be a key-player protein interacting with each of the EFC proteins (72), this unanimous lack of detection raises questions and the absence of its LSDV ortholog prompts us to wonder about a possible LSDV protein substitute. In contrast to O3, F9 orthologs are conserved throughout the POXV family, including CaPV. Excluding Zachertowska et al., for which deficiencies in MS instrumentation performance were reported (61), F9 and its orthologs are detected unanimously in poxvirus proteomics studies (Fig. 3; Table S2 in the supplemental material). This protein is selected in the study by Ngo et al. (40), whereas the LSDV024/F9 ortholog is detected but not selected in the current study (Table 2). Regarding these discrepancies, one specific feature of F9 deserves special attention. Indeed, the F9 protein has been demonstrated to be an EFC-associated protein, i.e., peripherally located, rather than a core EFC component (3). Interestingly, detection of this protein within the EFC, requiring highly sensitive techniques, is suggestive of small amounts interacting with EFC components in a non-stoichiometric or weak/unstable way (73). These features (peripheral association and unstable interaction) may have played some part in the differences observed between studies regarding this protein packaging. In so far as these properties could also be observed for the LSDV024/F9 ortholog, this could explain, to some extent, the variation observed in MS results which led us to not select F9 among candidates for packaging. In particular, in the current study, we could speculate that the peripheral location of LSDV024/F9 ortholog in the EFC may have increased its exposure to a proteolytic effect of trypsin during the purification process (74), resulting in the cleavage of the LSDV024/F9 ortholog and its removal from the virus preparations. However, it should be noted that these same observations cannot be made for the LSDV060/L1 ortholog, even though this protein is assumed to be also peripherally associated to EFC (3). Indeed, converging evidence has demonstrated L1 packaging in the MV VACV virion (39), and L1 is selected by Ngo et al. (40), and the LSDV060/L1 ortholog is selected in the current study. Nevertheless, to gain clarity on the lack of selection of F9, further investigations are clearly required, some of which could rely on the use of alternative proteolytic enzymes as well as on immuno-affinity purification techniques that have proven useful for vaccinia MV (73).

Among the 29 VACV proteins previously demonstrated as being part of the LSDV MV virion (39) and classified within the viral structure group, 19 proteins (61%) have their LSDV orthologs selected as constitutive of the LSDV MV virion (Table 2). Of the 29 proteins listed by Condit et al. (39), Ngo et al. selected 22 proteins (70%), one of which does not have an LSDV ortholog (A26).

In the group of selected LSDV proteins, 5 (LSDV031/F17 ortholog, LSDV101/A10 ortholog, LSDV094/A3 ortholog, LSDV105/A14 ortholog, and LSDV095/A4 ortholog) rank among the 10 most detected/abundant proteins in our experiment. These top 10 most abundant LSDV proteins represent a total of 54% of the total NSAF of the viral proteins (Table 1). Interestingly, their VACV orthologs are among the most abundant detected proteins comprising the VACV MV particle (56). Among these most abundant VACV packaged proteins, it makes sense to observe three major core proteins, namely A4, A3, and A10, being an integral part of the vaccinia virion core wall (3, 39).

One other protein, namely LSDV028 (Table 1), selected in the current study (Table 2), is the ortholog of the VACV F13 protein (75). The presence of this protein should be analyzed in parallel with the presence of the seven other membrane wrapping proteins (76), namely A56 (77, 78), F12 (79, 80), B5 (81, 82), A34 (83), A36 (84), A33 (85), and K2 (86). Among the wrapping membrane proteins, LSDV028/F13 ortholog was the only protein selected in the present study (Table 2), while a total of five wrapping membrane proteins (F12, B5, A34, A33, and F13) was detected (Fig. 3). Although not selecting any (Table 2), Ngo et al. (40) detected all eight wrapping membrane proteins (Fig. 3; Table S2 in the supplemental material). In other poxvirus proteomic studies (Fig. 3; Table S2 in the supplemental material), F13 (and its poxvirus orthologs) is unique in that it is the wrapping membrane protein which is most detected (Fig. 3: all studies except Zachertowska and Chung), whereas the detection of other wrapping membrane proteins varies between studies. Interestingly, we observe that the most recent studies, with the highest genome coverage (number of detected proteins over number of genomic ORFs), detected the eight wrapping proteins (Fig. 3; Table S2 in the supplemental material). Therefore, we might speculate that the extent of detection of the wrapping membrane proteins could be related to the sensitivity of the MS instrumentation used, allowing for the detection of residual wrapping membrane proteins derived from EV particles. This hypothesis could be considered plausible in the present study as EV particles were identified through a qualitative EM approach (Fig. 2). Alternatively, as suggested previously (59), the predominant F13 (and orthologs) detection could be due to an interaction of F13 with an MV surface protein, resulting in the presence of F13 on a minor subset of MV particles derived from disrupted EV, in agreement with the required involvement of F13 in MV wrapping (87). Further poxvirus proteomic investigations, using an EM approach that provides accurate quantification of EV particles, would contribute to a better understanding of the significance that the detection of wrapping membrane proteins could have.

Among the 29 VACV proteins listed by Condit et al. (39) and grouped in the virus structure category, it is noticeable that some key-player proteins do not have their LSDV ortholog selected.

Among these proteins of importance, our result regarding the LSDV A17 ortholog (LSDV109) may raise questions. As in the vast majority of the poxvirus proteomics studies for which A17 is detected (Fig. 3; Table S2 in the supplemental material), the LSDV109/A17 ortholog is also detected in our study (Table 2). However, while A17 is selected in the Ngo et al. study, the unstable detection of the LSDV ortholog in our experiment led us to not select it from the candidates for packaging (Table 2). This unstable detection of the LSDV109/A17 ortholog needs to be analyzed with regard to the stable detection of two other MV membrane proteins (LSDV117/A27 ortholog and LSDV105/A14 ortholog). Indeed, as observed with VACV A14 and A27 in the Ngo et al. study (Table 2), the LSDV117/A27 ortholog and LSDV105/A14 ortholog are both selected as candidates for packaging (Table 2). This observation is of particular interest, given that an interaction between these three MV membrane proteins is demonstrated for VACV. Indeed, A14 and A17 are two transmembrane proteins, spanning membrane twice, interacting with each other for the biogenesis of the vaccinia virion membrane (88). In addition, the integral membrane protein A17 anchors A27 via a cooperative binding mechanism (89 – 91). VACV envelope protein A27 binding to A17 affects two important biological stages: the virion assembly/egress stage and the infection pathway of virus progeny (endocytosis versus plasma membrane fusion) (91). Based on these known interactions, and in so far as they are similarly maintained between LSDV orthologs, not selecting the LSDV109/A17 ortholog when its molecular partners (LSDV117/A27 ortholog and LSDV105/A14 ortholog) are, is quite unexpected. Although further investigations are required, a plausible and simple explanation could rely on the specific physical and chemical properties of LSDV109/A17 orthologs (e.g., high hydrophobicity, glycosylation, and ionization profile) that may be distinct from A17 (38% of amino-acid sequence identity) and susceptible to making its detection more variable and challenging using classical shotgun proteomics approach.

Finally, two additional proteins to consider within the virus structure group are other membrane proteins, LSDV100/A9 ortholog and LSDV046/I5 ortholog, which are missing in our LSDV protein candidates selected for packaging. In fact, these two proteins were not detected at all inside the purified virus preparations in our study. Ngo et al. selected I5 (although detected only once in five replicates) and detected but did not select A9 (Table 2). In the other poxvirus proteomics studies (Fig. 3; Table S2 in the supplemental material), detection of these two proteins, although variable, remains prominent (Fig. 3; Table S2 in the supplemental material). While Chung et al. detected I5 and A9 among the low-abundance proteins (ranked 63rd and 69th, respectively) (56), CWPX A10/A9 ortholog is ranked among the 10 most abundant detected viral proteins (57). Considering this variability, we cannot rule out that either a low abundance or the physico-chemical properties of these proteins could explain the lack of detection observed in purified LSDV preparations and could have made their detection technically challenging. In the present study, considering that parameter settings were already specifically adjusted to target low-abundance proteins, another way to improve the detection of these two LSDV proteins could be to use enzymes other than trypsin (chymotrypsin, ArgC AspN, GluC) (92), which would offer different cleavage specificities and, therefore, additional possibilities to better identify proteins that would not have been detected in MS analysis of trypsin-only digested virus preparations.

Turning to VACV proteins listed by Condit et al. (39), comprising the viral transcription and genomic integrity groups, we observe that among 29 proteins, only 12 (41%) have their LSDV orthologs selected for packaging in the current study. The others either have their LSDV orthologs detected or undetected (E4), or have no identified LSDV orthologs (O2) (26, 27). Remarkably, Ngo et al. selected 28 proteins (96%) of the listed 29 VACV proteins (Table 2). A detailed examination of the multicomponent transcription apparatus may illustrate the difficulties met within this protein group. Considering all the poxvirus proteomics studies [with the exception of Zachertowscha et al., for which deficiencies in MS instrumentation performance were reported (61)], the eight subunits comprising the poxvirus DNA-dependent RNA polymerase were either unanimously detected (A5, A24, A29, J4, and J6) (Fig. 3; Table S2 in the supplemental material) or detected in most studies (E4, D7, G5.5, and their orthologs) (Fig. 3; Table S2 in the supplemental material). In the current study, three LSDV subunits (LSDV119/A29 ortholog, LSDV069/J4 ortholog, and LSDV071/J6 ortholog) are selected for packaging, while four subunits are only detected (LSDV096/A5 ortholog, LSDV116/A24 ortholog, LSDV085/D7, and LSDV055/G5.5 ortholog) and only one subunit was not detected (LSDV036/E4 ortholog). A similar observation is possible for the other components of the transcription apparatus either unanimously detected [excluding Zachertowska et al., for which deficiencies in MS instrumentation performance were reported (61)] or detected in most poxvirus proteomic studies (H6) (Fig. 3; Table S2 in the supplemental material). The LSDV orthologs of the two subunits of VACV early transcription factors (LSD084/D6 ortholog and LSDV098/A7 ortholog) and the LSDV ortholog of the VACV packaged DNA binding protein (LSDV043/I1 ortholog) were selected in our study as candidates for packaging. In contrast, the LSDV ortholog of the VACV poly (A) polymerase VP 55 (LSDV032/E1 ortholog), the LSDV ortholog of the VACV poly (A) polymerase small subunit VP39 (LSDV068/J3 ortholog), the LSDV orthologs of the VACV DNA helicases NPHI and NPHII (LSDV088/D11 ortholog and LSDV049/I8 ortholog), the LDV ortholog of the VACV topoisomerase H6 (LSDV077), and the LSDV ortholog of the VACV RNA polymerase-associated transcription-specificity factor RAP94 (LSDV75/H4 ortholog) were all detected but not selected as candidates for packaging. Excluding LSDV036/E4 ortholog which was not detected, all of these LSDV proteins were detected in this study, consistent with the detection of their poxvirus orthologs in the vast majority of proteomic studies on poxvirus (Fig. 3; Table S2 in the supplemental material). However, the observed variation in the level of detection of these LSDV proteins, which led to their exclusion from packaging candidates in the current study, calls for further investigations when we consider that many of these proteins play a key role during infection.

Finally, the last protein category regards the immunomodulatory effectors group. Actually, in the list of proteins demonstrated to be packaged into the VACV MV particle (39), Condit et al. identified only one single protein, H1, characterized as an immunomodulatory effector. Indeed, H1L codes for a dual-specificity phosphatase VH1 that down-regulates intracellular anti-viral response (93) and is released after viral entry from lateral bodies (LB) of the VACV particle (94, 95). H1 is detected unanimously among poxvirus proteomics studies [excluding the study on MYXV for which deficiencies in MS instrumentation performance were reported (61)] and is selected for packaging in the Ngo et al. study (40). The LSDV072/H1 ortholog is also selected in the current study (Table 2). Detected unanimously through poxvirus proteomics studies (Fig. 3; Table S2 in the supplemental material), two other components of VACV LB with immunomodulatory activities, phosphoprotein F17 and oxidoreductase G4, are either selected (F17) or only detected (G4) in the Ngo et al. study (Table 2). In the current study, LSDV031/F17 ortholog and LSDV053/G4 ortholog rank among the most abundant detected proteins (1st and 16th respectively) and were both selected (Table 2). The selection of LSDV031, LSDV053 ,and LSDV072 as candidates for packaging may suggest the existence of a close association between these three proteins as observed between their VACV F17, G4, and H1 orthologs and VACV particles (95), which could represent a preliminary indication of a possible LB residency of these proteins in LSDV MV particles. Moreover, four other LSDV proteins, namely LSDV144/A55, LSDV001/B15 ortholog, LSDV013/B16 ortholog, and LSDV007/C10 ortholog, were all selected in the current study. In contrast, there are no converging studies demonstrating packaging of the orthologs of these proteins in the VACV particle (39), and Ngo et al. did not select any of these proteins but detected all of them (Table 2). In proteomics studies, detection of these four protein orthologs seems quite variable, either between studies on VACV or between poxviruses (Fig. 3; Table S2 in the supplemental material). For these four proteins, one factor that may contribute to such a variable detection could be the low abundance of these proteins, as suggested by a consistent detection mainly in the most recent proteomic studies (Fig. 3; Table S2 in the supplemental material). Remarkably, this low abundance is also observed in the present study for LSDV144/A55, LSDV001/B15 ortholog, LSDV013/B16 ortholog, and LSDV007/C10 ortholog, which rank 110th, 82nd, 104th, and 89th, respectively, among the 111 proteins detected (Table 1). In the present study, detecting proteins with such low abundance supports the sensitivity of the experimental set-up used, including MS instrumentation as well as parameter settings. Clearly, the meaning that could be attributed to the packaging of this type of protein remains to be elucidated and further confirmatory work is needed. However, especially for this group of immunomodulatory effectors, differences between poxviruses of different genera (*Orthopoxvirus*, MYXV, and CaPV) should not be viewed as a totally unexpected result since we are analyzing distinct biological entities here, exhibiting, for instance, *in vitro* different host cell range requiring different intracellular modulators.

So, applying our selection method to all detected bovine proteins, a total of 65 bovine cell proteins persistently fell within the packaged region of the quantitation histogram in the five experiments (Table S2). Although some of these bovine cell proteins may be effectively packaged as previously evidenced (63, 64), the presence of these proteins could instead reflect residual contamination of MV preparations. This alternative hypothesis seems all the more likely since these proteins belong to classes previously described as having been associated with such types of contamination (40, 59, 65). Indeed, we cannot rule out a commonly observed contamination of purified virus preparations originating from proteins tightly bound to virions or associated with intracellular organelles (exosomes, mitochondria, nuclei, or other vesicles) which co-sediment with virus in both tartrate and sucrose gradient purification procedures (40, 65). In order to address properly the packaging of bovine proteins, it will be necessary to carry out experiments specifically designed to evidence cell proteins packaged into particles of enveloped viruses (95 – 98). The list of host proteins proposed here could possibly represent a starting point for designing additional studies specifically addressing this issue.

In conclusion, this study characterized for the first time the proteome of infectious viral MV particles of the LSDV KSGP-0240 strain. First, this analysis aims to participate in the better characterization of this viral strain, which remains, up to now, incomplete. We may therefore consider that our study brings a significant additional information layer to this strain characterization. However, it is clear that in order to provide a comprehensive insight into the LSDV proteome, that the same proteomic characterization deserves to be implemented on the other LSDV strains, with the newly emerged ones being of utmost interest. In particular, deciphering the proteomic profile of recombinant LSDV strains, recently evidenced for instance in Russia (99, 100) could help in better understanding the mosaic nature of their genome, including regions from both vaccine and virulent field LSDV strains. Such larger proteomic characterization of CaPV strains may be especially enlightening, in particular for the comparison between virulent and attenuated viral strains, for which we could get possible clues upon the determinants of CaPV virulence.

Finally, our study represents a first incursion into the proteome of CaPV, all the more informative since currently the vast majority of proteomics studies have focused on the unique genus of OPV, and especially on VACV. Indeed, the sole exception comprises the proteomics study of MYXV (61). We may therefore anticipate that providing new information about other chordopoxviruses will contribute to shedding new light on protein composition within the POXV family and bridge the proteome differences with the existing genetic differences between different genera of *Chordopoxvirinae*.

## SUPPLEMENTAL MATERIAL

Supplemental legends and tables jvi.00723-23-s0001.docx Tables S1 and S4 and legends of all supplemental tables.

Table S2 jvi.00723-23-s0002.xlsx Poxvirus proteomes.

Table S3 jvi.00723-23-s0003.xlsx Relative abundance of viral and host proteins.

ASM does not own the copyrights to Supplemental Material that may be linked to, or accessed through, an article. The authors have granted ASM a non-exclusive, world-wide license to publish the Supplemental Material files. Please contact the corresponding author directly for reuse.
