# Supplementary material for: Constitutive proteins of lumpy skin disease virion assessed by next-generation proteomics
Source: J Virol. 2023 Sep 22;97(10):e00723-23. doi: 10.1128/jvi.00723-23 (PMC10617387; doi:10.1128/jvi.00723-23)
Supplement: Supplemental legends and tables — Tables S1 and S4 and legends of all supplemental tables. [file jvi.00723-23-s0001.docx]

Constitutive proteins of lumpy skin disease virion assessed by next-generation proteomics

Léo Schlosser-Perrin^a^, Philippe Holzmuller^a^, Bernard Fernandez^a^, Guylaine Miotello^b^, Noureddine Dahmani^a^, Aymeric Neyret^c^, Stéphane Bertagnoli^d^, Jean Armengaud^b^, Philippe Caufour^a#^

^a^UMR ASTRE, CIRAD, INRAE, University of Montpellier (I-MUSE), Montpellier, France

^b^Département Médicaments et Technologies pour la Santé (DMTS), Université Paris Saclay, CEA, INRAE, Bagnols-sur-Cèze, France

^c^CEMIPAI, University of Montpellier, UAR3725 CNRS, Montpellier, France

^d^IHAP, Université de Toulouse, INRAE, ENVT, Toulouse, France

Running Head: Proteome of lumpy skin disease viral strain KSGP-0240 [54 characters and spaces]

# Address correspondence to Philippe Caufour, caufour@cirad.fr

**TABLE LEGENDS**

**Table S1. Characterization of replicates for each virion purification method (sucrose and tartrate).**

**Table S2. Proteome of vaccinia virus, monkeypox virus, cowpox virus, myxoma virus and lumpy skin disease virus. (Modified, with permission, from Ngo T, Mirzakhanyan Y, Moussatche N, Gershon PD. 2016. Protein primary structure of the vaccinia virion at increased resolution. J Virol 90:9905-9919.)**

**Table S3. Relative abundance (NSAF) of lumpy skin disease and host proteins identified in our viral preparations (sucrose and tartrate).**

**Table S4.**  Identified and selected host protein by LC-MS/MS. Data in Table S4 are compiled from five different LSDV preparations per condition, with two conditions (tartrate and sucrose continuous gradients). and represent host protein demonstrating reliably and systematically, for the five replicates of a given condition, values of the tartrate/sucrose NSAF ratio inside the defined limits. Only proteins with at least 2 peptides were counted.

|  |  |  |  |  |  |  |  |  |  |  |  |  |
| --- | --- | --- | --- | --- | --- | --- | --- | --- | --- | --- | --- | --- |
| Tests on viral stocks before purification |  |  |  |  |  |  |  |  |  |  |  |  |
| **Replicate N°** | **R1** | **R2** | **R3** | **R4** | **R5** |  |  |  |  |  |  |  |
| PCR P32 (Ct) | 19,8 | 19,2 | 21,2 | 18,9 | 19,5 |  |  |  |  |  |  |  |
| Viral titer (TCID50/ML) | 10^7.3^ | 10^7.5^ | 10^6.8^ | 10 ^8^ | 10^7.1^ |  |  |  |  |  |  |  |
|  |  |  |  |  |  |  |  |  |  |  |  |  |
|  |  |  |  |  |  |  |  |  |  |  |  |  |
|  |  |  |  |  |  |  |  |  |  |  |  |  |
|  | Sucrose banding method | | | | | mean | Tartrate banding method | | | | | mean |
| **Replicate N°** | **S1** | **S2** | **S3** | **S4** | **S5** |  | **T1** | **T2** | **T3** | **T4** | **T5** |  |
| PCR P32 (Ct) | 16,0 | 15,4 | 14,6 | 15,5 | 14,5 | 15,2 | 15,5 | 16,3 | 14,7 | 15,5 | 15,5 | 15,5 |
| **MS analyses** |  | | | | |  |  | | | | |  |
| Viral protein number | 105 | 103 | 110 | 107 | 106 | 106 | 109 | 108 | 108 | 110 | 105 | 108 |
| Bovine protein number | 1341 | 1240 | 1327 | 1393 | 1266 | 1313 | 1374 | 1172 | 1215 | 1206 | 1136 | 1221 |
| ratio [n° bovine protein / n° viral protein] | 12,8 | 12,0 | 12,1 | 13,0 | 11,9 | 12,4 | 12,6 | 10,9 | 11,3 | 11,0 | 10,8 | 11,3 |
| Viral protein NSAF (sum) | 88 | 70 | 105 | 87 | 102 | 91 | 98,9 | 120,93 | 115,39 | 119,25 | 112,33 | 113 |
| Bovine protein NSAF (sum) | 270 | 238 | 292 | 279 | 275 | 271 | 283 | 225 | 276 | 289 | 250 | 264 |
| Total NSAF | 358 | 308 | 397 | 366 | 377 | 361 | 382 | 346 | 391 | 408 | 362 | 378 |
| proportion of Viral protein NSAF/total NSAF | 25 | 23 | 27 | 24 | 27 | 25 | 26 | 35 | 29 | 29 | 31 | 30 |
| proportion of Viral protein NSAF/total NSAF | 75 | 77 | 73 | 76 | 73 | 75 | 74 | 65 | 71 | 71 | 69 | 70 |
|  |  |  |  |  |  |  |  |  |  |  |  |  |

**Supplemental Table S1**

**Supplemental Table S4**

| Category and functional description | Protein accession no.^a^ | Mass (d) | Specific peptides | Peptides total | Tartrate (NSAF) | Sucrose (NSAF) | Number of hits across replicates | Rank (relative abundance) | Proteins closely associated with other viruses |
| --- | --- | --- | --- | --- | --- | --- | --- | --- | --- |
| **Chaperone** |  |  |  |  |  |  |  |  |  |
| Heat shock protein 75 kDa, mitochondrial | Q2TBI4.1 | 79331 | 10 | 13 | 0,4412 | 0,3656 | 5 | 56 |  |
|  |  |  |  |  |  |  |  |  |  |
| **Transport** |  |  |  |  |  |  | 5 |  |  |
| ABC transporter TAP2 | AAL85633.1 | 79253 | 4 | 5 | 0,1514 | 0,1009 | 5 | 64 |  |
| Exportin-2 | A5D785.1 | 110305 | 14 | 23 | 0,6799 | 0,5258 |  | 53 |  |
| Solute carrier family 25, member 1 precursor, partial | DAA20380.1 | 30190 | 5 | 14 | 2,7824 | 1,9212 | 5 | 30 |  |
| Transporter 2, ATP-binding cassette, sub-family B | DAA16704.1 | 79210 | 2 | 6 | 0,1515 | 0,1136 | 5 | 63 |  |
| Tubulin alpha-1D chain | Q2HJ86.1 | 50251 | 3 | 35 | 12,9749 | 10,6665 | 5 | 3 |  |
| Tubulin beta-3 chain | Q2T9S0.1 | 50400 | 7 | 26 | 11,131 | 9,5833 | 5 | 5 |  |
| Tubulin beta-4B chain | Q3MHM5.1 | 49799 | 6 | 31 | 17,6911 | 14,3979 | 5 | 2 |  |
| Tubulin beta-5 chain | Q2KJD0.1 | 49639 | 6 | 31 | 18,6547 | 15,3911 | 5 | 1 |  |
| Tubulin, alpha 1 | AAX09051.1 | 49892 | 2 | 30 | 11,565 | 9,6007 | 5 | 4 | HCMV (64), VACV (56), ASFV (63) |
|  |  |  |  |  |  |  |  |  |  |
| **Translation** |  |  |  |  |  |  |  |  |  |
| 40S ribosomal protein S11 | Q3T0V4.3 | 18419 | 15 | 16 | 8,3066 | 5,8092 | 5 | 13 |  |
| 40S ribosomal protein S9 | A6QLG5.1 | 22578 | 18 | 20 | 9,6554 | 6,5108 | 5 | 10 |  |
| 60S ribosomal protein L10a | Q5E9E6.3 | 24816 | 5 | 18 | 9,8324 | 7,4549 | 5 | 7 |  |
| 60S ribosomal protein L13a | Q3SZ90.3 | 23508 | 12 | 16 | 3,0628 | 2,4672 | 5 | 26 |  |
| 60S ribosomal protein L14 | Q3T0U2.3 | 23421 | 8 | 9 | 2,9888 | 2,0494 | 5 | 27 |  |
| 60S ribosomal protein L18 | Q5E973.3 | 21522 | 11 | 13 | 7,4343 | 4,9717 | 5 | 15 |  |
| 60S ribosomal protein L9 | Q3SYR7.1 | 21863 | 5 | 18 | 5,1685 | 3,4305 | 5 | 18 |  |
| Ribosomal protein S14 | AAI02537.1 | 16766 | 2 | 5 | 2,8629 | 1,9086 | 5 | 29 |  |
| Ribosomal protein S2 | AAI02228.1 | 31216 | 19 | 21 | 9,5784 | 7,6243 | 5 | 8 |  |
| Ribosomal protein L9-like | DAA23756.1 | 21754 | 3 | 16 | 4,5509 | 3,0339 | 5 | 22 |  |
|  |  |  |  |  |  |  |  |  |  |
| **Signal transduction** |  |  |  |  |  |  |  |  |  |
| PREDICTED: ras-related protein Rab-2A isoform X3 | XP_005215509.1 | 23504 | 7 | 16 | 5,1906 | 3,4037 | 5 | 19 |  |
| Prohibitin-2 | Q2HJ97.1 | 33337 | 20 | 23 | 6,4193 | 4,2595 | 5 | 16 |  |
| RAB1A, member RAS oncogene family | ABM21543.1 | 22663 | 6 | 18 | 3,6624 | 2,6475 | 5 | 23 |  |
| RAB33B protein | AAI14732.1 | 25602 | 3 | 3 | 0,7031 | 0,4687 | 5 | 55 |  |
| Ras-related protein Rab-1B | Q2HJH2.1 | 22188 | 3 | 13 | 2,2535 | 1,5324 | 5 | 32 |  |
| Ras-related protein Rab-21; Flags: Precursor | Q17R06.1 | 24131 | 5 | 8 | 0,9531 | 0,5802 | 5 | 48 |  |
| Ovarian/breast septin gamma-like | DAA18161.1 | 74925 | 12 | 20 | 0,1869 | 0,1468 | 5 | 59 |  |
| Progesterone receptor membrane component 2 | DAA20848.1 | 23696 | 5 | 5 | 1,3504 | 1,0128 | 5 | 39 |  |
|  |  |  |  |  |  |  |  |  |  |
| **Metabolism** |  |  |  |  |  |  |  |  |  |
| ATP5J2 (ATPase) protein | AAI08209.1 | 10265 | 2 | 2 | 1,7535 | 1,5587 | 5 | 33 |  |
| Calcium-binding mitochondrial carrier protein SCaMC-1 | A5PJZ1.1 | 53250 | 18 | 18 | 1,1455 | 0,77 | 5 | 43 |  |
| Chain D, bovine mitochondrial F1-ATPase | 1BMF_D | 51673 | 26 | 31 | 8,0119 | 6,3863 | 5 | 12 |  |
| Chain D, low resolution structures of bovine mitochondrial F1-ATPase | 2W6E_D | 56249 | 27 | 31 | 7,3601 | 5,8668 | 5 | 14 |  |
| Chain G, ground state structure of F1-ATPase from bovine heart mitochondria | 2JDI_G | 30237 | 16 | 19 | 3,3403 | 2,3812 | 5 | 24 |  |
| Chain I, bovine heart cytochrome C oxidase at the fully oxidized state | 1V54_I | 8497 | 5 | 6 | 4,5899 | 4,1191 | 5 | 17 |  |
| Chain S, structure of the membrane extrinsic region of bovine ATP synthase | 2WSS_S | 20786 | 2 | 8 | 1,2508 | 0,8179 | 5 | 40 |  |
| Chain W, structure of mammalian respiratory complex I, class2 | 5LC5_W | 15044 | 3 | 3 | 0,8641 | 0,5318 | 5 | 51 |  |
| Cytochrome b5 type B (outer mitochondrial membrane) | DAA20034.1 | 16268 | 3 | 4 | 0,9835 | 0,7991 | 5 | 44 |  |
| Hexokinase domain containing 1 | DAA14289.1 | 102512 | 19 | 33 | 0,8975 | 0,5951 | 5 | 50 |  |
| Hydroxyacyl-coenzyme A dehydrogenase (trifunctional protein), beta subunit | AAI02639.1 | 51312 | 13 | 14 | 1,1303 | 0,8185 | 5 | 42 |  |
| Hydroxy-delta-5-steroid dehydrogenase, 3 beta- and steroid delta-isomerase 1 | AAI11204.2 | 42193 | 20 | 22 | 2,9389 | 1,9197 | 5 | 28 |  |
| NADH-cytochrome b5 reductase 3 | P07514.3 | 34100 | 15 | 16 | 3,3138 | 2,2874 | 5 | 25 |  |
| Receptor of activated protein C kinase 1 | P63243.3 | 35055 | 15 | 17 | 5,0777 | 3,4802 | 5 | 21 |  |
| Ribonucleoside-diphosphate reductase large subunit (RRM1) protein | AAI09487.2 | 89962 | 15 | 18 | 0,0778 | 0,0667 | 5 | 66 |  |
| Saccharopine dehydrogenase-like oxidoreductase | Q3T067.1 | 47289 | 3 | 4 | 0,1903 | 0,1269 | 5 | 60 |  |
| Thioredoxin-like 1 | DAA15792.1 | 32261 | 3 | 4 | 0,155 | 0,155 | 5 | 61 |  |
| Tyrosine 3-monooxygenase/tryptophan 5-monooxygenase activation protein, epsilon polypeptide | AAI02929.1 | 29155 | 11 | 19 | 1,5435 | 1,2691 | 5 | 36 |  |
| Voltage-dependent anion channel 1 | AAI02114.1 | 30821 | 13 | 26 | 8,5007 | 6,7162 | 5 | 11 |  |
| Voltage-dependent anion-selective channel protein 1 | DAA27459.1 | 30722 | 25 | 27 | 9,0489 | 7,1284 | 5 | 9 |  |
| Voltage-dependent anion-selective channel protein 2 (VDAC2) protein | AAI02905.1 | 31600 | 17 | 21 | 10,3481 | 7,6899 | 5 | 6 |  |
|  |  |  |  |  |  |  |  |  |  |
| **Other** |  |  |  |  |  |  |  |  |  |
| Brain protein 44 isoform 1 | DAA32047.1 | 14273 | 4 | 4 | 1,7516 | 1,0509 | 5 | 37 |  |
| Chromatin modifying protein 6 | AAI18186.1 | 23492 | 3 | 3 | 0,2128 | 0,2128 | 5 | 57 |  |
| DEAD (Asp-Glu-Ala-Asp) box polypeptide 3, X-linked | DAA12727.1 | 73083 | 3 | 24 | 0,9715 | 0,7663 | 5 | 45 |  |
| DEAD (Asp-Glu-Ala-Asp) box polypeptide 3, Y-linked | DAA12540.1 | 73110 | 2 | 19 | 0,8891 | 0,6839 | 5 | 47 |  |
| DEAD (Asp-Glu-Ala-Asp) box polypeptide 31 | DAA24143.1 | 80948 | 20 | 27 | 1,5442 | 1,0748 | 5 | 38 |  |
| Hydroxysteroid (17-beta) dehydrogenase 4 | AAI22585.1 | 79508 | 22 | 27 | 1,132 | 0,9056 | 5 | 41 |  |
| Lectin, mannose-binding 2 | DAA27641.1 | 40376 | 11 | 14 | 1,7832 | 1,3622 | 5 | 35 |  |
| Non-classical MHC class I antigen isoform 1 | DAA16412.1 | 40782 | 2 | 8 | 0,7111 | 0,5885 | 5 | 52 |  |
| Probable ATP-dependent RNA helicase DDX27 | A1A4H6.1 | 87033 | 2 | 2 | 0,0919 | 0,0804 | 5 | 65 |  |
| Rho/rac guanine nucleotide exchange factor (GEF) 2 | DAA31839.1 | 111753 | 5 | 7 | 0,1611 | 0,1163 | 5 | 62 |  |
| Serine palmitoyltransferase 1 | Q3MHG1.1 | 52755 | 8 | 9 | 0,6445 | 0,5308 | 5 | 54 |  |
|  |  |  |  |  |  |  |  |  |  |
| **Unknown** |  |  |  |  |  |  |  |  |  |
| Atlastin 3 | DAA13555.1 | 60273 | 13 | 14 | 0,8793 | 0,6968 | 5 | 46 |  |
| Hypothetical protein BOS_2447 | DAA32205.1 | 111329 | 11 | 12 | 0,2515 | 0,1707 | 5 | 58 |  |
| Transmembrane emp24 domain containing protein 9 | Q3T133.1 | 27281 | 7 | 9 | 2,6025 | 1,8328 | 5 | 31 |  |
| Transmembrane emp24 protein transport domain containing 7 | DAA25915.1 | 25320 | 7 | 8 | 1,7773 | 1,4218 | 5 | 34 |  |
| Transmembrane protein 33 | DAA28747.1 | 27966 | 5 | 7 | 0,7867 | 0,7152 | 5 | 49 |  |

^a^ Accession numbers are from the Genbank or Uniprot database

Abbreviations: AFSV, African swine fever virus; HCMV, human cytomegalovirus; VACV, vaccinia virus.
